# Molecular machineries of ciliogenesis, cell survival, and vasculogenesis are differentially expressed during regeneration in explants of the demosponge *Halichondria panicea*

**DOI:** 10.1186/s12864-022-09035-0

**Published:** 2022-12-29

**Authors:** Ana Riesgo, Nadia Santodomingo, Vasiliki Koutsouveli, Lars Kumala, Michelle M. Leger, Sally P. Leys, Peter Funch

**Affiliations:** 1 Department of Biodiversity and Evolutionary Biology, Museo Nacional de Ciencias Naturales (CSIC), Calle José Gutiérrez Abascal 2, 28006 Madrid, Spain; 2 Department of Life Sciences, Natural History Museum, Cromwell Road, London, SW5 7BD UK; 3 Department of Earth Sciences, Oxford University, South Parks Road, Oxford, OX1 3AN UK; 4 Marine Symbioses Research Unit, GEOMAR Helmholtz Centre for Ocean Research Kiel, Düsternbrooker Weg 20, D-24105 Kiel, Germany; 5 Nordcee, Department of Biology, University of Southern Denmark, Campusvej 55, 5230 Odense M, Denmark; 6 Marine Biological Research Center, University of Southern Denmark, Hindsholmvej 11, 5300 Kerteminde, Denmark; 7 Institute of Evolutionary Biology (CSIC-UPF), Paseo Marítimo de la Barceloneta 37-49, 08003 Barcelona, Spain; 8 Department of Biological Sciences, University of Alberta, 11455 Saskatchewan Drive, Edmonton, Alberta T6G 2R3 Canada; 9 Department of Biology, Aarhus University, Ny Munkegade, 114-116 Aarhus C, Denmark

**Keywords:** Porifera, Transcriptomics, Regeneration, Angiogenesis

## Abstract

Sponges are interesting animal models for regeneration studies, since even from dissociated cells, they are able to regenerate completely. In particular, explants are model systems that can be applied to many sponge species, since small fragments of sponges can regenerate all elements of the adult, including the oscula and the ability to pump water. The morphological aspects of regeneration in sponges are relatively well known, but the molecular machinery is only now starting to be elucidated for some sponge species. Here, we have used an explant system of the demosponge *Halichondria panicea* to understand the molecular machinery deployed during regeneration of the aquiferous system. We sequenced the transcriptomes of four replicates of the 5–day explant without an osculum (NOE), four replicates of the 17–18–day explant with a single osculum and pumping activity (PE) and also four replicates of field–collected individuals with regular pumping activity (PA), and performed differential gene expression analysis. We also described the morphology of NOE and PE samples using light and electron microscopy. Our results showed a highly disorganised mesohyl and disarranged aquiferous system in NOE that is coupled with upregulated pathways of ciliogenesis, organisation of the ECM, and cell proliferation and survival. Once the osculum is formed, genes involved in “response to stimulus in other organisms” were upregulated. Interestingly, the main molecular machinery of vasculogenesis described in vertebrates was activated during the regeneration of the aquiferous system. Notably, vasculogenesis markers were upregulated when the tissue was disorganised and about to start forming canals (NOE) and angiogenic stimulators and ECM remodelling machineries were differentially expressed once the aquiferous system was in place (PE and PA). Our results are fundamental to better understanding the molecular mechanisms involved in the formation of the aquiferous system in sponges, and its similarities with the early onset of blood-vessel formation in animal evolution.

**Supplementary Information:**

The online version contains supplementary material available at 10.1186/s12864-022-09035-0.

## Introduction

Morphogenesis and regeneration are central aspects of animal biology since these are the fundamental processes by which a tissue, organ, or organism develops its shape. The ability to regenerate a lost body part is relatively common across the animal tree of life, but whole-body regeneration (WBR) is less frequent [1, 2]. Epimorphosis involves regenerating from a blastema of undifferentiated cells and usually includes proliferative cellular stages, like the regeneration of a limb, while morphallaxis regenerates the tissue with pre-existing cells and no cell proliferation, like it occurs in *Hydra* [3]. Besides epimorphosis and morphallaxis, metaplasia is another regenerative mechanism that involves transdifferentiation of cells [4], although this mechanism is considered rare in the animal kingdom [1]. However, the boundaries between epimorphic, morphallactic and metaplastic regeneration are often difficult to establish, with some organisms showing features of both, and many authors tend to avoid this terminology [5]. Because regeneration has important implications for modern medicine, much research has been carried out, advancing our understanding of the physiological and molecular underpinnings of the process. From the resulting large body of research, common patterns are starting to emerge: such as re-epithelization; involvement of matrix metalloproteinases, enzymes to degrade or remodel the extracellular matrix; bioelectric events (e.g. ion fluxes or membrane voltage changes) and immune responses triggered at the onset of regeneration; cell signaling pathways, especially Wnt and fibroblast growth factor (FGF) signaling; and the role of innervation [1]. Whole-body regeneration is considered to be the ancestral mode of regeneration, and it has its most striking examples in sponges, ctenophores, cnidarians, and placozoans [1, 6].

Sponges (phylum Porifera) are fundamental to understanding the origins of basic biological processes because even though they show relative molecular complexity, they have fewer specialised cell types than most other phyla, and these retain a pluripotency unparalleled in almost all other phyla [7–9]. But also, they are key because of their crucial position among the earliest diverging animals (see [10, 11] for the latest analyses). As mentioned, sponges possess a large body of stem or pluripotent cells, which are behind their enormous regeneration capacities [9]. Indeed, in sponges, all types of regeneration have been described [6], but the most striking among them is perhaps the ability to undergo somatic embryogenesis (or formation of an individual using “somatic” cells) after complete dissociation into component cell types, often also referred to as reaggregation [6, 12–14]. Remarkably, even from cell cultures containing only archaeocytes, sponges are able to regenerate almost all tissues and layers of adult sponges in less than 24 hours, highlighting the totipotent nature of archaeocytes [15]. Similarly, when using larval cell cultures containing mostly archaeocytes, the sponges are formed within 3–4 days [16]. However, when the cell culture contains mostly choanocytes, they never reaggregate to reorganise a functional sponge [17, 18].

During their life, sponges undergo shape shifts, regeneration of body parts, cell transdifferentiation, and budding almost routinely, but they are also capable of whole-body regeneration, although this is most likely only occurring in experimental conditions. Such regenerative plasticity has been coined as “chronic morphogenesis” [6], and it is at the core of the ecological and evolutionary success of sponges. The morphological basis of sponge regeneration patterns have been studied profusely in the last two centuries, from first reports in the early twentieth century [19], to complex descriptions of the process in the recent years [5, 6, 14, 20, 21]. Sponge regeneration can go through epithelial–to–mesenchymal transformations, where epithelial cells (mostly pinacocytes and choanocytes) transdifferentiate into other cell types to regenerate the sponge body, and mesenchymal–to–epithelial transformations, where archaeocytes and other somatic cells are the ones transdifferentiating into epithelial cells (reviewed in Ereskovsky et al., 2021). However, the molecular aspects of all these types of sponge regeneration are definitely less well-known [22–25].

**Fig. 1:**
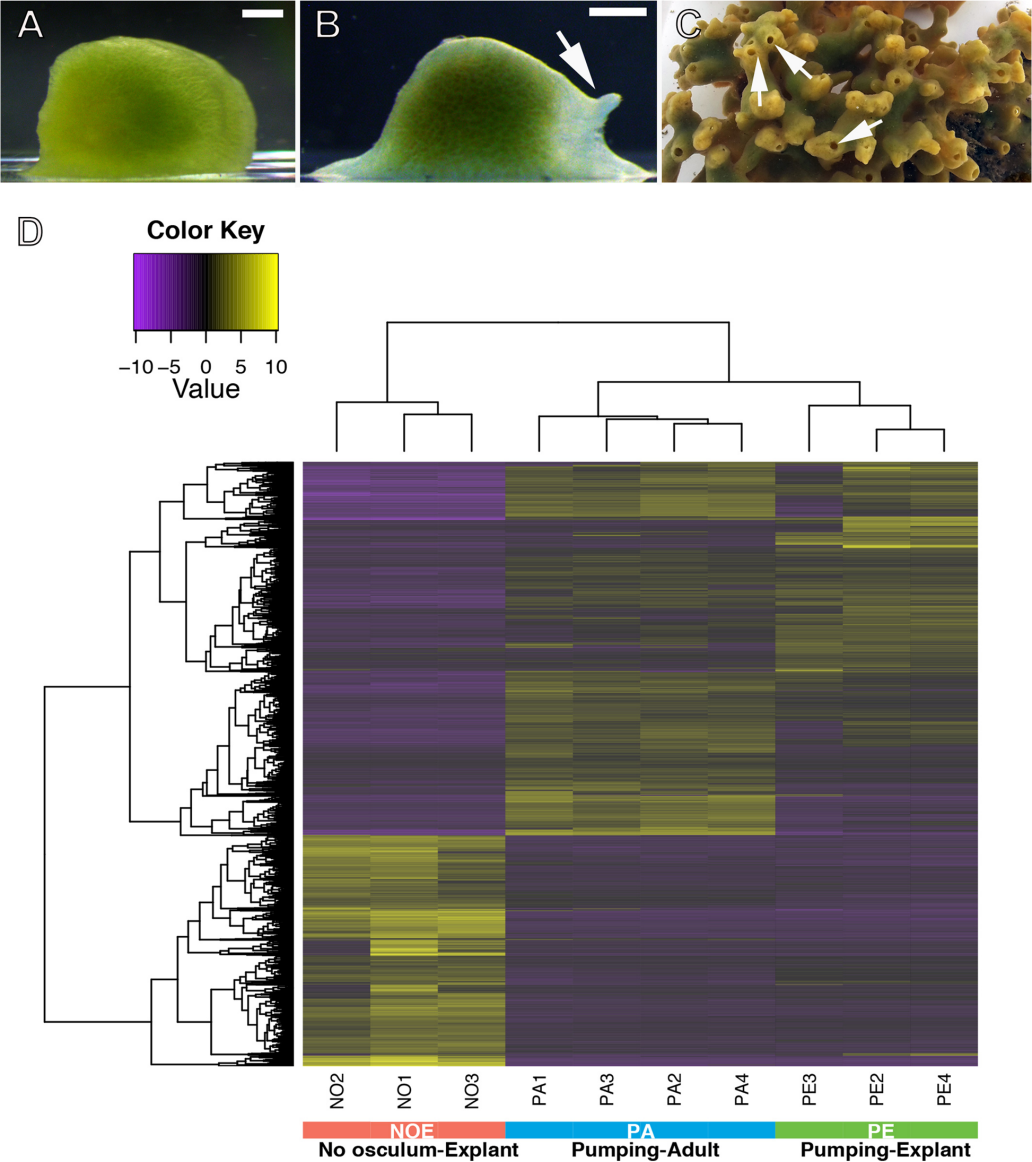
Explant experimental design and results of the gene expression analysis during aquiferous system remodeling in *Halichondria panicea*. **a** Five-day explant without osculum (NOE). **b** Explant with osculum (arrow) and pumping activity (PE). **c** Field-collected adult sponge with pumping activity (PA). **d** Heatmap showing differential gene expression among the three conditions of the experiment: NOE, PE and PA

*Halichondria panicea* is a common intertidal sponge in the North Atlantic Ocean, which has previously been the subject of regeneration studies characterising the morphological processes during the regeneration of the sponge body (“explant”) from small body fragments of parent sponges [26, 27]. Usually, the initial explant contains all or most tissue elements of the parent sponge, including epithelia, choanoderm, mesohyl with extracellular matrix (ECM), and the pinacoderm lining the aquiferous system [6]. During the regenerative process, the explants rearrange the polarity of their body in order to position their osculum in the direction of the flow [28]. The main steps of regeneration in the explants are: epithelization of the wound surfaces, disintegration of the aquiferous system, cell de-differentiation, loss of polarity and symmetry, attachment to the substrate, reestablishment of polarity, and finally restoration of the aquiferous system including the formation of the exhalant opening known as the osculum [6, 28]. The molecular aspects of this complex process of regeneration are poorly known, with descriptions of the process in just a few sponge species so far [14, 23, 25], hampering our understanding of the transcriptional processes behind regeneration in such a plastic animal. Here, we address such dearth of molecular data on regenerative processes in sponges by using an RNAseq approach in *H. panicea* during explant regeneration, including two steps of the process: once epithelization is complete but polarity, osculum, and aquiferous system are absent (NOE) and once the aquiferous system is reformed, the polarity re-established, and the explant is pumping (PE). We compared the expression levels of the whole transcriptome between these two stages and also to intact sponges with full pumping activity (PA) living in their natural habitat (Fig. 1A-C). Our results are discussed in the context of existing literature of regeneration in sponges and other animals with extensive regenerative capacities.

## Materials and methods

### Sampling and explant formation

Intact, actively water-pumping adult specimens (PA) of the demosponge *Halichondria panicea* with widely open exhalant openings were collected from the inlet to Kerteminde Fjord, Denmark (55°26′59″N, 10°39′40″E), and were instantly processed into explants at the nearby Marine Biological Research Center. Explants were obtained from cuttings (5–10 mm^3^), which were prepared and cultivated according to [28]. Cuttings adhered to glass slides in flow-through aquaria with aerated bio-filtered seawater (15–25 psu, 15 °C), and regenerated their epithelial exopinacoderm within 3–5 days, as seen from smoothing of the explants’ periphery and edges (Fig. 1A). We defined these stages as ‘no-osculum explants’ (NOE), in which an osculum and thus, a fully functional water canal system is not yet developed. The formation of a venting osculum in attached explants during the next 15–18 days indicated remodelling of the internal aquiferous system and filter-feeding (Fig. 1B). These stages were defined as ‘explants with a single osculum and pumping activity’ (PE). Active water pumping in PE, NOE and PA was tested by adding fluorescein dye (Sigma-Aldrich) to the sponge surface prior to sampling for histological and transcriptomic analyses. PE explants were fed with *Rhodomonas salina* algae and a mix of bacteria that grew within the algal culture.

### Light and Electron microscopy

Samples of NOE (*n* = 4), PE (*n* = 4), and PA (*n* = 4) were fixed in formalin 4% and another batch in 2.5% glutaraldehyde in 0.4 M PBS + 0.34 M NaCl (pH 7.4, at 25 °C) for histological examination and detailed observation of the tissue organisation. Formalin-preserved samples were rinsed in distilled water while glutaraldehyde-preserved samples were rinsed in 0.4 M PBS + 0.6 M NaCl for at least 1 hour, followed by the removal of the spicule’s silica in 5% hydrofluoric acid in distilled water over 5 hours, and then rinsed in distilled water until further processing.

For light microscopy, formalin-preserved samples were dehydrated through a series of ethanol of increasing concentration from 70 to 100%. Samples were then soaked in xylene for 30 min and embedded in paraffin at 58 °C overnight. Sections 5 μm thick were cut using a Microm HM 325 Rotary microtome (ThermoFisher Scientific) and stained using standard hematoxylin-eosin or toluidine blue protocols. In 10 sections of each sample, we quantified the number of archaeocytes, amboeboid cells, and choanocytes (free and in chambers) per 0.01mm^2^.

For electron microscopy, samples were postfixed in 1% osmium tetroxide and dehydrated through an ethanol series of increasing concentration from 70 to 100%. For Scanning Electron Microscopy (SEM), samples were further critical point-dried, carbon-coated (20 nm), and imaged in a Zeiss Ultra Plus Field Emission Electron Microscope at the Natural History Museum of London (NHM). For Transmission Electron Microscopy (TEM), samples were embedded in Spurr resin for at least 3 days. Thin sections were cut with an ULTRACUT-T Leica ultramicrotome, stained with 2% lead citrate and 2% uranyl acetate and observed with a Hitachi H-7650 TEM at Kew Gardens, operating at 80 kV.

### RNA extraction and RNAseq library preparation

Total RNA was extracted using a standard Trizol-based method using TRI Reagent (Life Sciences) following the manufacturer’s instructions. Subsequent mRNA purification was performed with a Dynabeads mRNA Purification Kit (Invitrogen) also following the manufacturer’s protocol. Twelve different cDNA libraries (NOE = 4, PE = 4, PA = 4) were constructed with the ScriptSeq v2 kit (Illumina), and sequenced alongside other samples in a single flowcell of Illumina NextSeq 500, at 150 bp paired-end read length.

### Transcriptome assembly and annotation

We first checked the original quality of the raw reads with FastQC (Babraham Bioinformatics) and processed all the libraries to remove adaptor sequences. Trimmomatic [29] was used to remove areas of sequence with low Phred scores (below 28), smaller than 30 base pairs, and complete sequences with overall low quality. Only the paired reads resulting from the trimming process were used to construct a de novo reference transcriptome using Trinity 2.4.0 [30] with all the default options. Raw reads can be accessed at the Short Read Archive (SRA) under Bioproject number PRJNA374707 (Accession numbers SRR1857509-SRR1857520). Completeness of the reference transcriptome was obtained using BUSCO [31] and the conserved eukaryotic and metazoan gene complements.

To annotate our transcripts, we used DIAMOND [32] against two different databases, i.e. Refseq and Swissprot (last accessed in September 2020), and retained only the best hit with an e-value threshold of 10^− 5^ for both cases. To obtain Gene Ontology (GO) terms, we ran Blast2GO PRO [33] and used the gene identities obtained both for Refseq and Swissprot separately, and collected the GO terms for Biological Process, Molecular Function and Cellular Component with the GOSlim function.

### Differential gene expression

The expression levels for the genes of all samples from different stages (NOE, PE, and PA) were obtained by mapping the clean reads (Additional file 1: Supplementary Table 1) from individual replicates to the reference assembly using RSEM [34] within the Trinity module [30]. We used the count table (for the genes) to further analyse the differential (pairwise) comparative expression of the three stages using the Bioconductor package edgeR [35] as part of the Trinity module [30]. We selected only those differentially expressed genes (DEG) with fold change over 2 and corrected *p*-values < 0.0001.

We performed a GO enrichment analysis with Blast2GO using the DGEs for each of the conditions against the whole reference transcriptome and plotted only those with a corrected *p*-value of 0.001. In addition, we used REVIGO [36] to visualise the main GO term categories associated with those DGEs obtained with edgeR. Finally, we also constructed gene networks of the DGEs in Genemania [37].

### Phylogenetic analyses

We performed separate phylogenetic analyses of the fibroblast and vascular endothelial growth factor receptors (FGFR and VEGFR respectively), tubulin (alpha, beta and delta), and myosins present in vertebrates and invertebrates, including sponges (see Additional file 2: Supplementary Fig. 3). We used the translated protein from the transcripts that blasted against these genes in *H. panicea* and aligned them with other orthologs from vertebrates and invertebrates (see Additional file 2: Supplementary Fig. 3) using the MUSCLE [38] aligner in SeaView [39] and then selecting for conserved regions with Gblocks 0.91b [40]. Phylogenetic inference with Maximum Likelihood was performed using RAxML-NG 1.0.3 [41].

## Results and discussion

### *Morphological changes during tissue regeneration in Halichondria panicea explants*

**Fig. 2:**
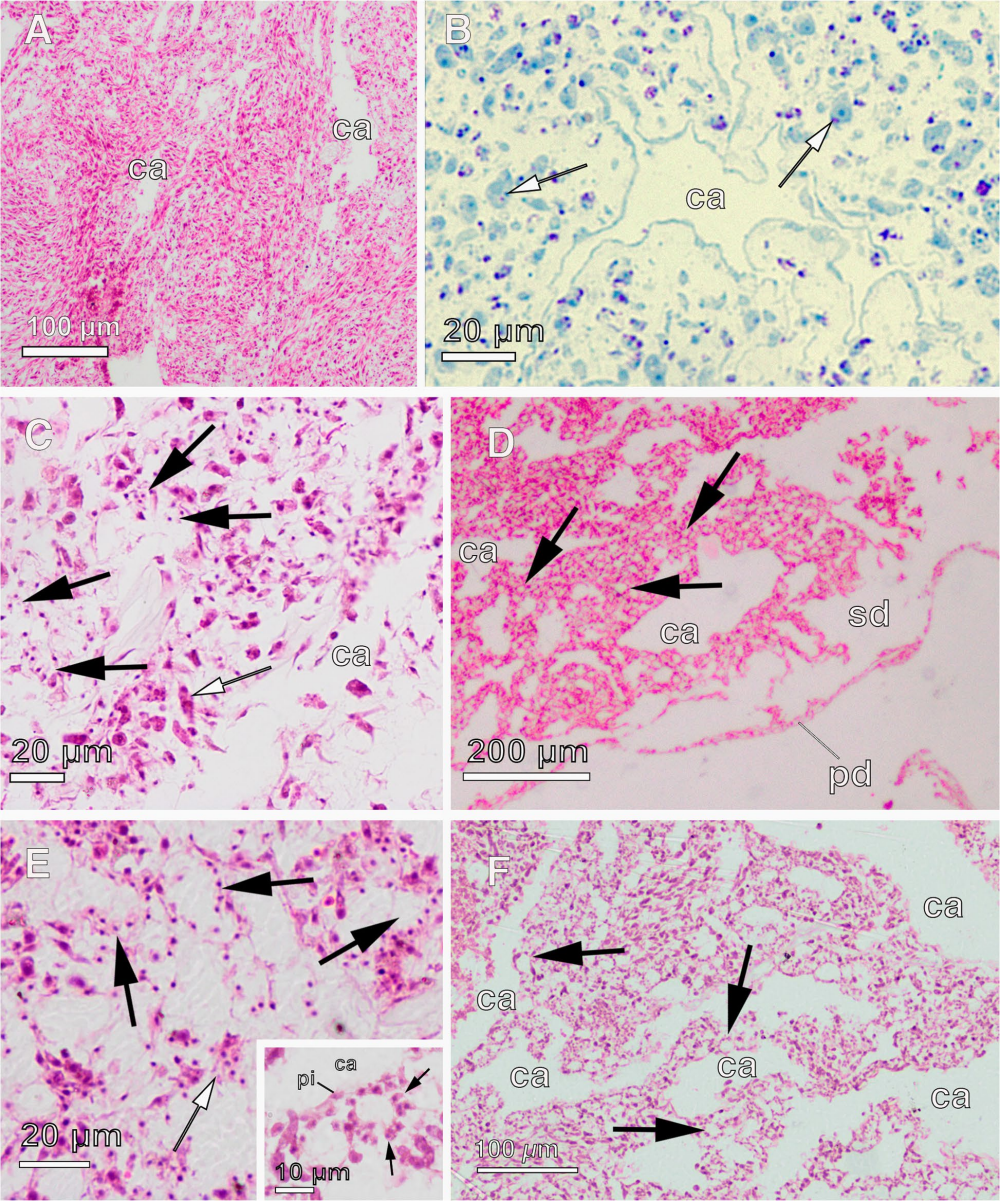
Histological sections of the aquiferous system of *Halichondria panicea* using light microscopy **a** No osculum explant (NOE) with a few intact canals (ca). **b** No osculum explant (NOE) with an intact canal (ca). White arrows indicate archaeocytes. **c** Pumping explant (PE) showing choanocytes aggregated and forming chambers in the mesohyl (black arrows) and archaeocytes (white arrow) close to canals (ca). **d** Pumping explant (PE) with a fully organized aquiferous system, including pinacoderm layer (pd), subdermal membrane (sd), canals (ca), and choanocyte chambers (black arrows). **e** Pumping explant (PE) showing choanocyte chambers (black arrows) and some aggregates of choanocytes (white arrows). Inset: Close up of a choanocyte chamber (black arrows) located near two canals (ca) lined by pinacocytes (pi). **f** Pumping explant (PE) with fully organized aquiferous system: choanocyte chambers (black arrows) and canals (ca)

**Fig. 3:**
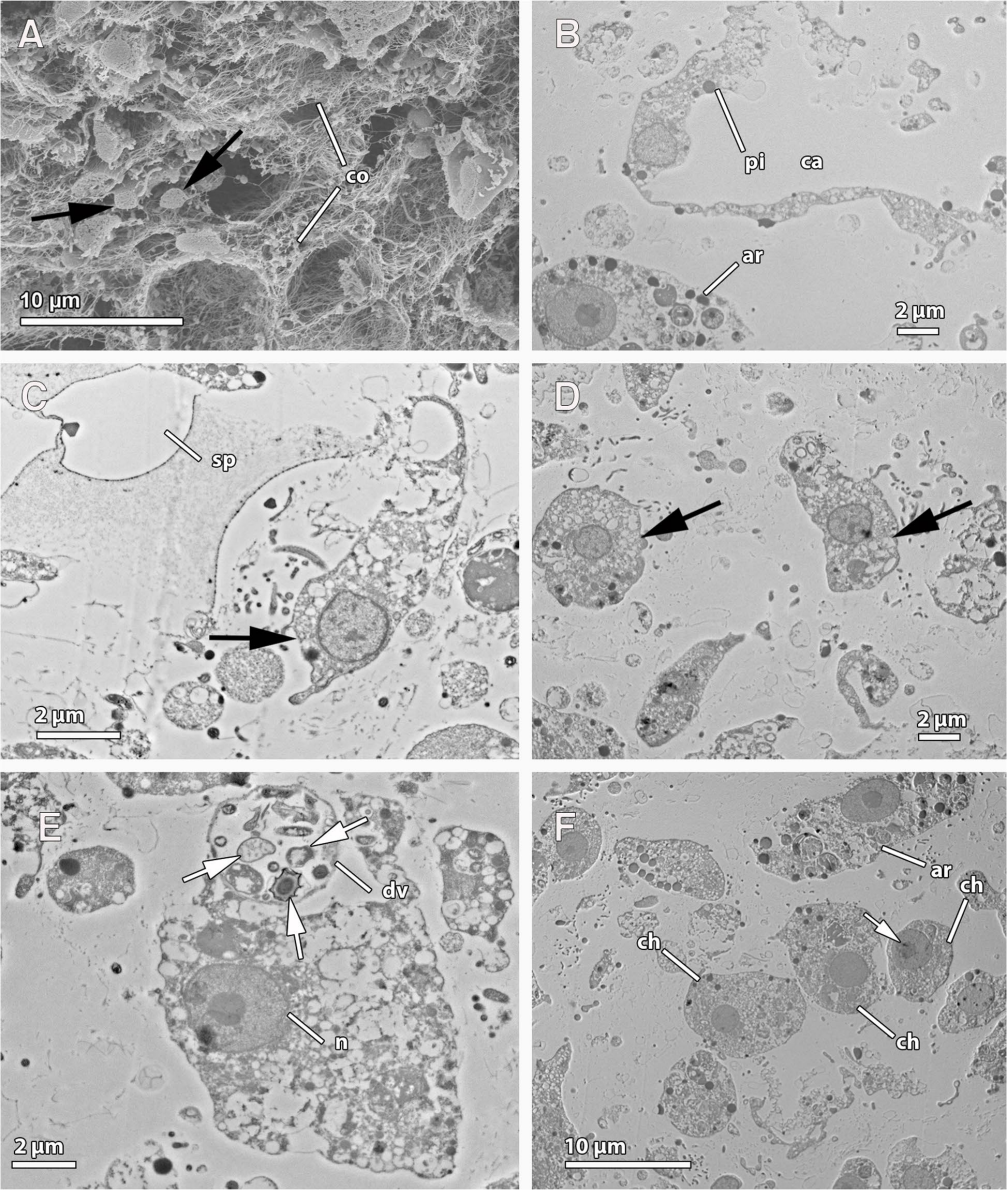
Ultrastructure of ‘no-osculum explants’ (NOE) with electron microscopy. **a** SEM micrograph showing disarrayed choanocyte chambers (black arrows) and abundance of collagen (co). **b** Canal (ca) lined by a flat pinacocyte (pi) with an archaeocyte (ar) in the vicinity. **c** Single choanocyte (black arrow) from a disarrayed chamber with the flagellum oriented towards the mesohyl where spicules lie (sp). **d** Disarrayed choanocytes (black arrows) in the mesohyl. **e** Archaeocyte with digestive vesicle (dv) containing several types of prokaryotes (white arrows), and a nucleolated nucleus (n). **f** Choanocyte chamber organized with choanocytes (ch) in close contact. Note the absence of flagella and archaeocytes (ar) in the vicinity and the nucleolated nucleus in one of the choanocytes (white arrow)

We observed extensive tissue regeneration during the transition of explants from ‘no-osculum’(NOE) to ‘water pumping’ stages with an osculum (PE; Fig. 1A-B). In NOE, chambers were disassembled, as seen by individual choanocytes in the mesohyl (Figs. 2A–C and 3A, C–D, F), some of them without a flagellum, which may have been disarranged. The aquiferous system was almost completely disarranged (Figs. 2A–B, 3B), although few canals seemed to remain intact in the mesohyl. Alternatively, these canals might have been newly formed. All 3 NOE individuals lacked subdermal cavities, the exopinacoderm was immediately followed by a high-density cellular organisation of the mesohyl, with a high degree of cell compaction (Fig. 2A), and it completely lacked choanocyte chambers or only few disarranged choanocyte chambers (Fig. 2A–B, 3C–D). The structural organisation of adult sponges (PA), previously described by Reiswig [42] and Barthel [43] and highly similar to PE sponges (Fig. 2D–F), was completely different to the disorganised tissue we found in NOE (Fig. 2A, 3A). During tissue remodelling, NOE sponges had mostly archaeocytes, amoeboid cells in the mesohyl (Fig. 3A–B, E–F), and few choanocytes, both with (Fig. 3C) and without a flagellum (Fig. 3D; Additional file 3: Supplementary Table 4). Choanocytes without flagella still retained similar features as those with the flagellum, including anucleolated nucleus and digestive vesicles, although some showed a larger cell body and a nucleolus (Fig. 3F). Such high numbers of archaeocytes and ameboid cells were not observed in PE and PA sponges (Additional file 3: Supplementary Table 4). In PE explants, choanocytes were observed in clusters or forming new chambers in the mesohyl (Fig. 2C). Also at the PE stage, an osculum and subdermal cavities were present (Fig. 2D), along with complete functional choanocyte chambers (Fig. 2E–F), and an aquiferous system including interconnected in- and excurrent canals (Fig. 2F).

### Assembly, mapping and differential gene expression

We obtained 327 million raw reads that were subsequently trimmed (Additional file 1: Supplementary Table 1). The reference assembly of *Halichondria panicea* was constructed with approximately 245 million clean reads (Additional file 4: Supplementary Fig. 1A) and resulted in 208,269 assembled transcripts (N50 1047 nt, see Additional file 1: Supplementary Table 1), corresponding to 67,844 protein-coding genes. Our BUSCO analysis recovered 99% of the eukaryotic conserved cassette and 96.1% of the metazoan cassette (Additional file 1: Supplementary Table 1). Between 30 and 78% of the clean reads were mapped uniquely to the reference transcriptome per sample (Additional file 1: Supplementary Table 1). Only 37% of the assembled sequences obtained a diamond hit against the refseq database, and 99% of those with a diamond hit had associated GO terms (Additional file 1: Supplementary Table 1).

Two samples (NOE_rep4 and PE_rep1) were discarded because their expression pattern was extremely divergent with respect to the other samples of the same stage (Additional file 2: Supplementary Figs. 2). These disparities were probably due to different timings of sampling and/or developmental stages during the experimental design. Three samples from NOE (NOE_rep1, 2, and 3) were cultured in May, 2016, whilst NOE_rep4 was cultured in September, 2016. It is possible that the sponges sampled for the preparation of explants in May were in a different physiological state than those collected in September, which lies within the reproductive period of this species in the same collection area [44]. Thus, sampling of our *H. panicea* sponges during the reproductive season (autumn) may have added too much noise to the analysis. Furthermore, we also observed a much faster development of the osculum in explant PE_rep1 (~ 10 days) compared to explants PE_rep2, PE_rep3 and PE_rep4 (17–18 days), which could explain the different expression patterns between these samples (Additional file 5: Supplementary Fig. 2). Removing NOE_rep4 and PE_rep1 resulted in more similar expression levels among samples in each stage (Additional file 4: Supplementary Fig. 1B–C).

A total of 1051 transcripts were upregulated (only 293 with significant diamond hit ID against the databases, hereafter referred as hit ID) and 638 were downregulated (only 261 with hit ID) in PE vs. NOE (Fig. 1D, Additional file 6: Supplementary Table 2). When comparing NOE vs. PA, 1328 transcripts were upregulated (only 243 with hit ID) in PA and 1121 transcripts downregulated (only 488 with hit ID) in PA (Additional file 6: Supplementary Table 2). In contrast, only 91 transcripts were upregulated (only 24 with hit ID) in PE when compared to PA, and 312 downregulated (only 48 with blast ID) (Additional file 6: Supplementary Table 2).

### Changes in gene expression during tissue regeneration

#### Ciliogenesis and ECM remodelling in NOE

**Fig. 4:**
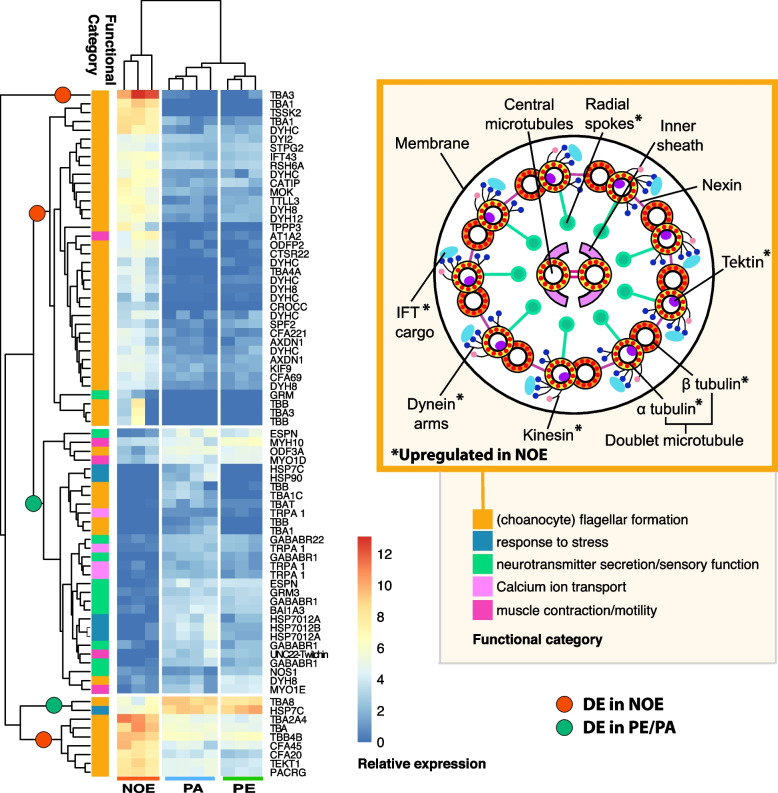
Differentially expressed genes related to ciliogenesis, stress, contractility, and sensory functions. Genes names are abbreviated and full names can be found in Additional file 7: Supplementary Fig. 4. GO terms are shown on the left side with parent categories on the right. The detailed structure of the flagellum is shown on the right side of the figure

In demosponges, transdifferentiation, including both epithelial–to–mesenchymal (EMT) and mesenchymal–to–epithelial (MET) morphogenesis, is the most widespread regenerative processes [6], with pinacocytes and choanocytes (flagellated cells) acting as main cell sources in the EMT morphogenesis and mostly archaeocytes transdifferentiating during MET morphogenesis. Although cell proliferation increases progressively during regeneration in other sponge species [21, 22], such process seems not to be the central mechanism involved in the regeneration of the wounds. Here in the NOE stage of *H. panicea*, we notably observed a massive decrease in canal structures and almost no choanocyte chambers (Additional file 3: Supplementary Table 4). The few choanocytes we observed lacked flagella and were part of disarranged chambers or free (Figs. 2A–B, 3A–C; Additional file 3: Supplementary Table 4). In addition, in NOE samples, we noticed a high density of archaeocytes (Fig. 2A–B) containing numerous digestive vesicles (Fig. 3E–F). Indeed, there were twice the number of archaeocytes per mm^2^ in the tissue of NOE individuals compared to both PE and PA (Additional file 3: Supplementary Table 4). In contrast, in PE samples, we found chambers with flagellated choanocytes and canals lined with flattened pinacocytes (Fig. 2C–D), indicating the progressive reconstruction of the aquiferous system. Furthermore, in PE, the formation of an osculum (Fig. 1A) indicated the presence of a fully developed aquiferous system (Fig. 3C-D), confirmed by our observation of active water-pumping in these explants [28, 45]. It remains unresolved if the choanocytes without flagellum observed in our morphological study of NOE (Fig. 3D, F) migrate through the mesohyl until they find chambers to join and then they rebuild their flagellum in the PE stages, like previously shown in explants of *Spongilla lacustris* [46], or if chambers in PE consist of entirely ‘new’ choanocytes which differentiated from a single or a conundrum of archaeocytes like it occurs during metamorphosis or asexual reproduction in other sponges (e.g., [47, 48]). However, assembly and disassembly of the flagellum is quite a common phenomenon in all organisms, from single-cell algae and sponges to mammals [49]. In *Chlamydomonas reinhardtii*, for instance, many environmental stimuli can trigger the disassembly of the flagellum [50]. Loss of flagellum could either be achieved by either resorption or deflagellation, which is in fact triggered by stress in many invertebrates [51]. In *Aplysina cavernicola* and *Oscarella lobularis*, during metaplasic regeneration, new flagella were formed in old choanocytes using their basal bodies which were not completely resorbed [5, 21]. In our RNASeq results, we found highly enriched functions in NOE compared to PE related to cilium formation, including polymeric cytoskeletal fibre, cilium, motile cilium, ciliary part, microtubule, cilium assembly, cilium organisation, cell projection assembly, and microtubule-based process (Additional file 4: Supplementary Fig. 1D). Many genes responsible for the correct formation and function of the flagellum, as previously observed in proteotranscriptomic studies of ciliogenesis of flagellated unicellular eukaryotes (reviewed in [47]), were upregulated in NOE, including *tubulin-α* and *tubulin-ß*, *dyneins*, *outer dense fiber 2*, *radial spoke head 6*, *rootletin*, *cilia- and flagella-associated proteins*, and *kinesin KIF9* (Fig. 4, Additional file 7: Supplementary Fig. 4). Kinesin and dynein proteins are important for intraflagellar transport (IFT) (Fig. 4 Additional file 6: Supplementary Table 2, Additional file 7: Supplementary Fig. 4), while tubulins are the main structural proteins of the flagellum microtubules; radial spoke heads are connecting proteins between the outer and the inner microtubules, and rootletins are part of the anchoring system and fundamental for maintenance of the flagellum [52]. Interestingly, during the course of flagellar assembly, α- and ß-tubulins are upregulated in the model system *Chlamydomonas reinhardtii*, but return to basal levels once the flagellum is formed [52]. It is important to note here, that while several transcripts of *tubulin-α* and *tubulin-ß* were upregulated in PE and PA, other transcripts with the same annotation were upregulated instead in NOE (Fig. 4, Additional file 6: Supplementary Table 2, Additional file 2: Supplementary Fig. 3C). Other genes related to the structure and maintenance of the flagellum were also found upregulated in NOE, such as *MAPK MAK MRK overlapping kinase* (*MOK*) which negatively regulates cilium length [53], and *tektin-1*, which is involved in the stability and structural complexity of the flagellum [54].

Many other genes traditionally related to the maturation of sperm and capacitation were also upregulated, such as *parkin*, *testis-specific serine threonine- kinases 1 and 2*, and *cation channel sperm-associated 2* [55]. Overall, these molecular and morphological results indicate that flagella are formed in choanocytes in NOE, regardless of them being newly formed choanocytes (indicating cell proliferation) or pre-existing cells without flagella, as observed in our morphological analysis of NOE.

**Fig. 5:**
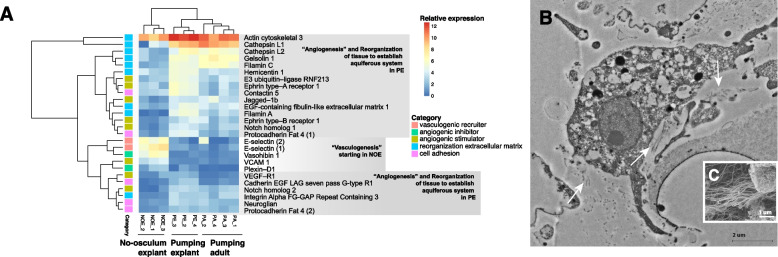
**a** Differentially expressed genes related to angiogenesis/vasculogenesis and ECM organization. GO terms are shown on the left side with parent categories on the right. **b** TEM micrograph of a spongocyte secreting collagen fibers (white arrows). *C. SEM* micrograph of a spongocyte secreting collagen fibers

Besides ciliogenesis, the synthesis and deposition of the extracellular matrix (ECM) are fundamental to maintain the tissue polarity and function. Many markers of ECM deployment were upregulated in NOE (Additional file 4: Supplementary Fig. 1E and Additional file 5: Supplementary Fig. 2), but others were engaged later (in PE) when reorganisation of the tissues and the aquiferous system was completed (Fig. 5). This is similar to the regeneration processes described in other demosponge species, such as *O. lobularis* and *H. caerulea* [5, 23], where ECM condensation and reorganisation occur very early in the process (sometimes only 2–3 hr. after injury) but can extend until the wound is completely healed.

#### Cell proliferation and cell survival

**Fig. 6:**
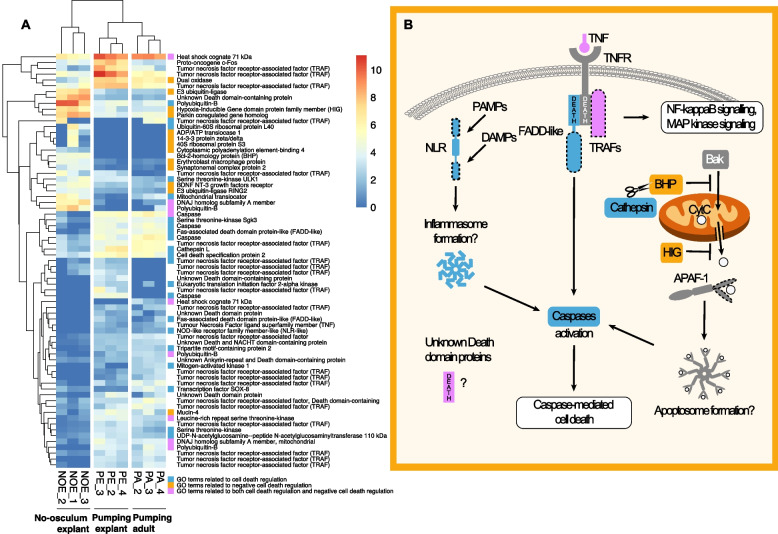
Differentially expressed cell death-related genes in *Halichondria panicea*. **a** Heatmap showing differentially expressed genes with predicted functions in regulated cell death, and GO terms associated with cell death (blue), its negative regulation (yellow), or both (pink) (Additional file 6: Supplementary Table 2). **b** Schematic figure showing selected products of differentially expressed genes mapped onto regulated cell death pathways. Blue, pathway components linked to regulated cell death induction in sponges and/or other animals. Yellow, pathway components linked to regulated cell death inhibition. Pink, pathway components linked to both induction and inhibition of regulated cell death. Grey, pathway components encoded in the *H. panicea* transcriptome, but not differentially expressed in our study. Dotted lines indicate partial sequences that lack information about specific domains in *H. panicea* that are found in vertebrate homologs. APAF-1, Apoptotic protease activating factor 1; Bak, Bcl-2 homologous antagonist/killer; BHP, Bcl-2 homologous protein; DAMPs, damage-associated molecular patterns; DEATH, Death fold motif found in some proteins implicated in regulated cell death and inflammation; FADD-like, Fas-associated death domain protein-like; HIG, hypoxia-inducible domain-containing protein; MAMPs, microbe-associated molecular patterns; MAPK, Mitogen-activated protein kinase; NF-κB, Nuclear factor κ-light-chain-enhancer of activated B cells; TNF, Tumour necrosis factor; NLR-like, NOD-like receptor; TNFR, Tumour necrosis factor receptor; TRAFs, Tumor necrosis factor receptor-associated factors

Interestingly, the GO categories (within Biological Process) most frequent among the DE genes in the pumping explant’ stage (PE) were ‘response to ethanol’ and ‘macrophage differentiation’ when compared to the ‘no-osculum explant’ stage (NOE; Fig. 6, Additional file 4: Supplementary Fig. 1E), and ‘apoptotic signaling pathway’ when compared to the intact, actively pumping sponges (PA) collected from the field (Fig. 6, Additional file 4: Supplementary Fig. 1F, J–I); this latter category contains mostly subcategories related to immune responses and cell death or survival. Similarly, during regeneration in the demosponge *Halisarca caerulea*, apoptotic processes were found to be fundamental for regenerating injured tissues [23, 25]. Within these groups of DE genes, we identified a number of homologs of genes that have been linked to regulated cell death in sponges and other animals.

Despite their position as the possible sister group to all animals, sponges possess many of the same characteristic genes implicated in regulating cell death processes in other animals. These include the apoptotic initiator and executioner proteases known as *caspases*; pro- and anti-apoptotic Bcl-2 family members, which trigger or block the release of mitochondrial cytochrome C upstream of caspase activation in intrinsic apoptosis; and tumour necrosis factor receptor (TNFR) family members [56], which trigger caspase activation through the action of adapter molecules in extrinsic apoptosis (Fig. 6, Additional file 6: Supplementary Table 2 and Additional file 8: Supplementary Table 3).

In sponges, regeneration often involves cell proliferation and migration of the stem cell pool (archaeocytes and choanocytes), but transdifferentiation has also been described [5, 6, 57, 58]. Here, we have identified several markers of cell proliferation that appear to be upregulated during relatively early phases of regeneration (NOE) (Additional file 6: Supplementary Table 2, Additional file 4: Supplementary Fig. 1G). These include genes involved in nuclear division as well as *TNF receptor-associated factor 5* and *Nicotinamide phosphoribosyltransferase*, which play important roles in cell proliferation in vascular tissues in other metazoans [59, 60]. Several caspases were downregulated in NOE relative to PE and PA, as was the inflammasome component *NOD-like receptor family member* (*NLR*), which detects microbe- or cellular stress-associated molecules and initiates inflammatory responses via caspase activation [61]. In addition, we identified a gene downregulated in NOE that exhibited overall sequence similarity to the bilaterian *Fas-associated death domain* (*FADD*) genes; FADD proteins interact with members of the Tumour Necrosis Factor Receptor (TNFR) family (which was also downregulated in NOE) and activate downstream apoptotic caspases. Other sponges encode the pro-apoptotic protein DD2, which comprises two death domains and has sequence similarity to both FADDs and the Fas ligand [62]. The FADD-like sequence encoded by the *Halichondria panicea* transcriptome was partial, such that we could not determine whether it presents the domain architecture of DD2 or of the FADDs found in other animals.

Consistent with these findings, genes upregulated in NOE included several proteins implicated in cell survival, such as the *apoptosis-suppressing erythroblast macrophage protein* (*EMP*) [63], a member of the hypoxia-inducible domain-containing protein family, which promotes cell survival under anoxic conditions [64], and the *anti-apoptotic Bcl-2-homologous protein* (*BHP*) [65, 66]. *Cathepsin L*, which, in human cells, may inhibit the BHP by cleaving it [67], was also downregulated in NOE.

Several unknown proteins containing death domains, which in bilaterians mediate protein-protein interactions in a subset of proteins that function in cell death and immunity, were identified in the *Halichondria panicea* transcriptome. Consistent with the diverse functions of death domain proteins in promoting either cell death or survival [68], many of these proteins were upregulated in PA and PE compared to NOE, while one was upregulated in NOE (Fig. 6, Additional file 6: Supplementary Tables 2 and Additional file 8: Supplementary Tables 3). Similarly, the TNF receptor-associated factor (TRAF) family includes both positive and negative regulators of cell death and innate immunity in bilaterian model organisms [69, 70], with some members functioning in promoting either survival or death depending on cleavage status [71]. Some TRAFs were found upregulated (*n* = 5) or while others were downregulated (*n* = 15) in NOE (Fig. 6, Additional file 6: Supplementary Tables 2 and Additional file 8: Supplementary Tables 3). Interestingly, during the later regenerative phase in *Halisarca caerulea*, *TNF receptor factor 3* was found to be upregulated [23].

Taken together, our results suggest that at the point when explants have regenerated their epithelia, i.e. 3–5 days after dissection, they exhibit increased expression of genes linked to cell survival, and repression of genes linked to cell death. This is consistent with earlier reports of apoptosis taking place following dissection in the demosponges *Aplysina cavernicola* [21] and *Halisarca caerulea* [23]. In those sponges, two waves of apoptosis take place, 6–12 hours and 48–72 hours post-operation in *A. cavernicola* and 2 hours and 12 hours post-operation in *H. caerulea*. Thus, in these examples both waves of apoptosis are complete before the time at which our NOE were sampled, consistent with a shift towards a pro-survival expression profile such as the one we report here.

#### Remodelling of the aquiferous system: Vasculogenesis- and angiogenesis-like processes in explant regeneration

Sponge morphology and body plan are phylogenetically constrained but they are also modulated to a certain extent by the environment [72–74]. For instance, changes in the water flow can induce reorganisation of the aquiferous system to improve filter-feeding and oxygen supply [75]. One of the most dramatic events promoting body remodelling in sponges is the removal of the osculum (where the exhalant current is released), which is considered as the primary organiser of the body plan in sponges [75–78]. In the absence of an osculum, such as in our NOE stage, the aquiferous system experiences a profound reorganisation until water flow is re-established and the current leaves the sponge body through a newly formed osculum. Interestingly, during the regeneration of the aquiferous system in *H. panicea* in our experiments, several genes of the molecular toolkit involved in vertebrate blood vessel formation were found upregulated (Fig. 5). As mentioned before, such regeneration of the aquiferous system included choanocyte chamber, canal and osculum formation processes. In vertebrates, blood vessels are formed through two processes called vasculogenesis, or de novo formation of endothelial cells, and angiogenesis, in which new vessels sprout from endothelial cells in pre-existing vessels [79]. Hypoxia is the primary physiological trigger for vessel formation, and this process is regulated first by chemotaxis and recruitment of endothelial cells during vasculogenesis, followed by angiogenesis regulated via both stimulators and inhibitors [80]. In general, vertebrate quiescent endothelial cells become activated by angiogenic signals (VEGF, growth factors, cytokines and chemokines [NO_PRINTED_FORM]), which results in degradation of the basement membrane, proliferation and migration of endothelial cells towards the angiogenic stimulus [81, 82]. These endothelial cells finally form a new basement membrane as well as ECM, and recruit perivascular cells to form a new, functional vessel [82] .

In our experiments, genes with important roles in the formation of new blood vessels (or vasculogenesis), such as *vascular cell adhesion molecule 1* and *E-selectin* [83], were differentially upregulated in NOE (Fig. 5). These chemokine proteins attract endothelial cells to start the formation of new blood vessels [83, 84]. Among the vertebrate stimulators of both vasculogenesis and angiogenesis, the most important are the *vascular endothelial growth factor receptor 1*, i.e. *VEGF-R1* [85], together with *ephrin* and the ephrin ligands [86]. In turn, both *vasohibin-1* and the interplay between *semaphorin* and *plexin-D1* count among the inhibitors of the sprouting process [79, 87]. In marine demosponges, flagellar activity and fluid movement (or pressure differentials in the water canal system) act as stimuli for formation and modelling of the excurrent canals [88]. The aquiferous system of canals in sponges is far from being homologous to the vertebrate vessels, mainly (but not only) because sponges do not transport blood in their aquiferous system but environmental water. However, it is not unreasonable to think that the formation of tubular structures to canalise fluids in metazoans is similar in its molecular regulation and hypoxia–related triggering mechanisms across phyla. In fact, sponges such as our single-osculum *H. panicea* explants, regularly experience internal hypoxia, and even anoxia [89–92], due to contractions of the body including the osculum and water canal system [45, 93]. Also, regenerating explants without a functional aquiferous system, are unable to supply their interior with oxygen and nutrients via water-pumping, leaving major parts of the explant depleted of oxygen [89]. Indeed, we found both *VEGF-R1* (Additional file 2: Supplementary Fig. 3A-B) as well as the angiogenic factors *ephrin type B receptor 1* and *ephrin type B receptor 2* more expressed (although not significantly) in NOE and PE explants than in PA, where the canals have been already well-formed (Fig. 5). In addition, the angiogenic inhibitor factors *vasohibin-1* and *plexin-D1* were differentially upregulated in NOE, when the canals were mostly disassembled (Figs. 2A–B, 5). It is important to note that although ephrin receptors are widely spread within sponges, their ligand, ephrin, has yet to be identified [94].

Other genes encoding proteins involved in signalling vessel formation through the Notch pathway, such as *jagged 1* and *galectin 3* [95] were differentially overexpressed in PE (Fig. 5). Although their role in the formation of the sponge aquiferous system is completely unknown, perhaps this whole array of stimulators and inhibitors of blood vessel formation have an ancestral role in positioning and forming canalicular systems in metazoans. Interestingly, even though invertebrates lack endothelial cells, the VEGF and VEGF-R molecules have proved to play a role in blood vessel formation in many invertebrate species [81, 82]. Furthermore, the VEGF-like factor of the nematode *Caenorhabditis elegans* is even able to induce angiogenesis in human cell cultures [96], suggesting the ancestral origin and high conservation of the mechanism. Although we cannot spatially resolve the expression of vascular genes in our experiments, the fact that the molecular machinery for vasculogenesis and angiogenesis is upregulated during remodelling of the aquiferous system in *H. panicea* explants is suggestive of a role in these processes.

In vertebrates, vasculogenesis and angiogenesis are accompanied by a reorganisation of the extracellular matrix (ECM), cell adhesion processes in the vessels, and formation of the basement membrane lining the vessels [NO_PRINTED_FORM]. During our explant regeneration experiments, we found evidence of a profound reorganisation of the tissue related to the synthesis and deposition of the ECM at both the morphological (Figs. 2–3) and molecular levels (Fig. 5, and Additional file 4: Supplementary Fig. 1E). In fact, collencytes, which are specialised cells secreting collagen, were often observed in NOE (Fig. 5B). Interestingly, genes involved in reorganisation of the ECM during angiogenesis, such as *integrin* and *filamin* [97], were upregulated in NOE. Furthermore, we found ubiquitous genes in septate junctions, i.e. *neuroglian* and *contactin*, upregulated once regeneration has been mostly completed in (PE), as well as in non-manipulated sponges (PA) (Fig. 5A).

Finally, one of the main aspects related to hemal circulatory systems in invertebrates is the general contractility of the vessels, usually accomplished by myoepithelial cells [81, 82]. In *Halichondria panicea*, periods of osculum closure and body contractions have been observed in actively pumping explants [93]. The aquiferous system of *H. panicea* explants seems to be contractile in response to both GABA and L-glutamate stimuli [45], as in other demosponge species [78, 98, 99]. Indeed, in sponges, the Rho/ROCK pathway, its downstream components, and the myosin regulatory light chain (MRLC) of myosin II, which mediate actomyosin contractility [100, 101], regulate the correct formation of the aquiferous system [102]. In our study, *myosin* genes were highly upregulated in stages with an (already formed) aquiferous system, i.e. PE and PA (Fig. 4) and were found to belong to myosin I and II families (Additional file 2: Supplementary Fig. 3C). Also, the gene *twitchin*, which, coupled with actin and myosin, is fundamental for the catch phase of contractions in molluscs [103], was highly upregulated in PE (Fig. 4, Additional file 6: Supplementary Table 2). Although we cannot resolve spatially the expression of these myosin genes, they are known to be expressed in pinacocytes lining the water canals and, to some extent, in archaeocytes, as previously shown in single-cell transcriptomic studies [104].

#### Response to stimulus once the osculum is formed

Sponge contractility and in general, sponge responses, are mostly triggered by amino acid signaling molecules [105]. In pumping explants (PE), GO categories involved in environmental stimuli sensing, including G-protein coupled receptor (GPCR) signalling pathways, response to biotic and external stimulus, response to other organisms, and transmembrane signalling receptor activity, were highly enriched (Additional file 4: Supplementary Fig. 1D, H). In our DE dataset, we found heat shock proteins, GABA receptors, mGluRs, and nitric oxide synthase, upregulated in PE and PA compared to NOE (Fig. 4). Similarly, both GPCRs and GABA receptors were upregulated upon recovery from mechanical damage and grazing in the marine sponge *Aplysina aerophoba* [25]. In addition, other genes involved in sensing the environment, like several homologs of *transient receptor potential cation channel subfamily A member 1* (*TRPA*), were upregulated in sponges with an aquiferous system, both PE and PA in contrast to NOE (Fig. 4). This family of transient receptor potential ion channels is involved in several processes of environmental sensing, including chemical and temperature signals as well as mechanical stress [106]. In general, environmental receptors including metabotropic glutamate receptors (mGluRs), GABA receptors, and G–protein coupled receptors, are highly diversified in sponges [107]. Interestingly, TRPAs are highly expressed in choanocytes, while mGluRs and GABARs are more expressed in pinacocytes [104]. What is most interesting here, is that our data indicates that the sensory activity usually developed by the aquiferous system in sponges [78, 108, 109] is clearly upregulated when the aquiferous system is fully formed.

## Conclusions

Recent comprehensive studies that combined morphological and molecular approaches, concluded that regeneration after complete dissociation, or whole–body regeneration, mimics post–larval development, at least in the calcareous sponge *Sycon ciliatum* [110]. Several pathways were common to both processes, including TGF-B, Notch, FGF, EGF, Hedgehog, and Wnt pathways [110], which play a prominent role in the regeneration of other invertebrates [111] and wound healing in sponges [23, 25]. Interestingly, during regeneration from a wound injury in the demosponges *Halisarca caerulea* and complete dissociation in *H. dujardini*, these classical developmental pathways had a central role in the regeneration of the wounded tissue [14, 23]. However, our results of regeneration in *Halichondria panicea* demosponge explants paint an alternative transcriptional picture, with ciliogenetic, angiogenic, and apoptotic molecular machineries playing the most important roles in the regenerative processes. Several of these pathways were also found to be important in *S. ciliatum*, *A. aerophoba*, and *H. caerulea* though, including apoptosis and remodelling of the ECM [23, 25, 110]. But it is important to note that our study was performed 3–5 days after explant formation, and classic developmental pathways may have played roles in earlier stages of the regeneration, which were not covered in our experiments. Our study demonstrates a shift towards pro-survival expression profiles after explant attachment/reformation of outer epithelia, including the formation of the internal water canal system. Furthermore, our findings indicate that the presence of an intact, i.e. functional aquiferous system (as observed in our PE and PA stages) coincides with an upregulation of genes for contractile behaviour, probably in response to environmental stimuli, although further research is needed to understand whether contraction processes occur in the absence of a fully formed aquiferous system. Both the similarities and the differences in the molecular processes involved in regenerative mechanisms of sponges strongly suggest a very plastic genomic regulation of processes during regeneration. The phylogenetic constraints of regeneration in sponges may be dictating the molecular events necessary to remove damaged cells and rebuild the tissues: while deploying the post-larval development machinery could be fundamental for some sponge species, others, such as our *H. panicea* explants, may use an alternative strategy to achieve body polarity and the formation of a fully functional aquiferous system. But it is important to note that the timing of sampling during regeneration is key for comparing expression profiles and morphogenetic movements, and for this reason comparing our data to other datasets in the current literature is difficult. There are many future directions we see stemming from our study that may enable a more holistic assessment of this fascinating regenerative processes in sponges, including the following: 1. Comparative research across sponge taxa and the same experimental conditions, 2. Detailed studies on the specific timing of tissue morphogenesis for components such as the epithelium and the choanocyte chamber formation, and 3. Subtractive analysis of processes that might be occurring normally but not involving classic regeneration, such as natural cell proliferation, contraction, etc.

### Supplementary Information

**Additional file 1.**

**Additional file 2.**

**Additional file 3.**

**Additional file 4.**

**Additional file 5.**

**Additional file 6.**

**Additional file 7.**

**Additional file 8.**

## Data Availability

Raw reads can be accessed at the Short Read Archive (SRA) under Bioproject number number PRJNA374707 (Accession numbers SRR1857509-SRR1857520).
