# Supplementary material for: Molecular machineries of ciliogenesis, cell survival, and vasculogenesis are differentially expressed during regeneration in explants of the demosponge Halichondria panicea
Source: BMC Genomics. 2022 Dec 29;23:858. doi: 10.1186/s12864-022-09035-0 (PMC9798719; doi:10.1186/s12864-022-09035-0)
Supplement: Supplementary file 2 — Additional file 2. [file 12864_2022_9035_MOESM2_ESM.pdf]

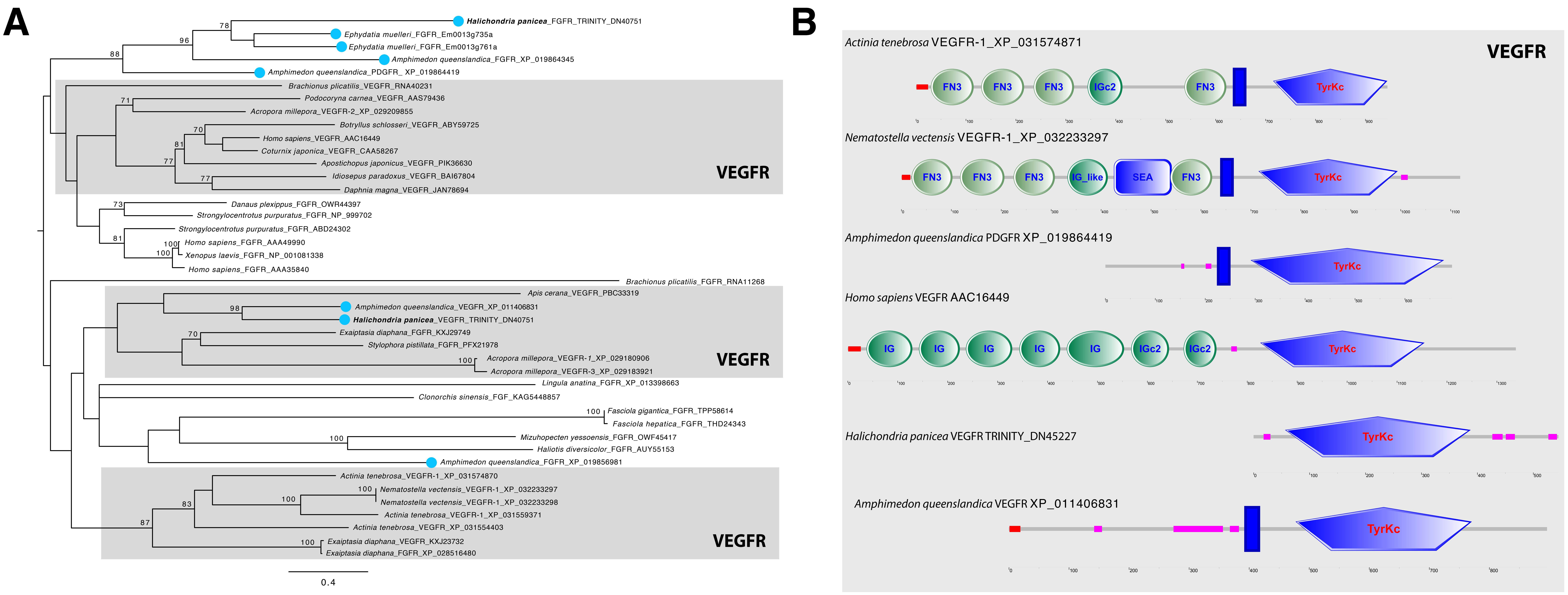

Supplementary Figure 3. A. VEGFR phylogenetic reconstruction using RAxML.
B. Domain structure of VEGFR in sponges and other invertebrates obtained in SMART.
C. Myosin phylogenetic reconstruction using RAxML.
D. Tubulin phylogenetic reconstruction using RAxML. Note that only node bootstrap support over 70 is shown.

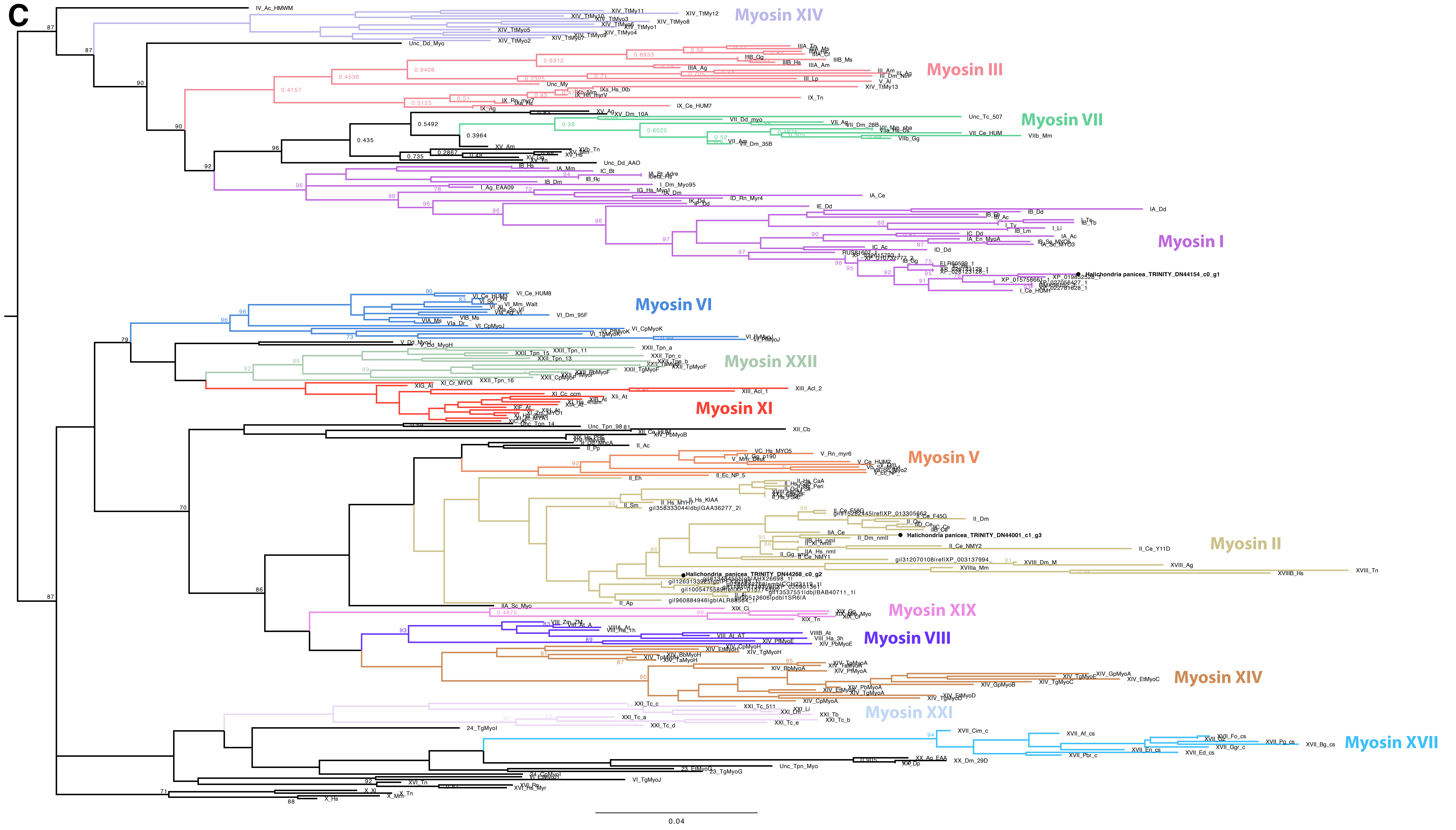
C
Myosin XIV
Myosin III
Myosin VII
Myosin I
Myosin VI
Myosin XXII
Myosin XI
Myosin V
Myosin II
Myosin XIX
Myosin VIII
Myosin XIV
Myosin XXI
Myosin XVII
0.04

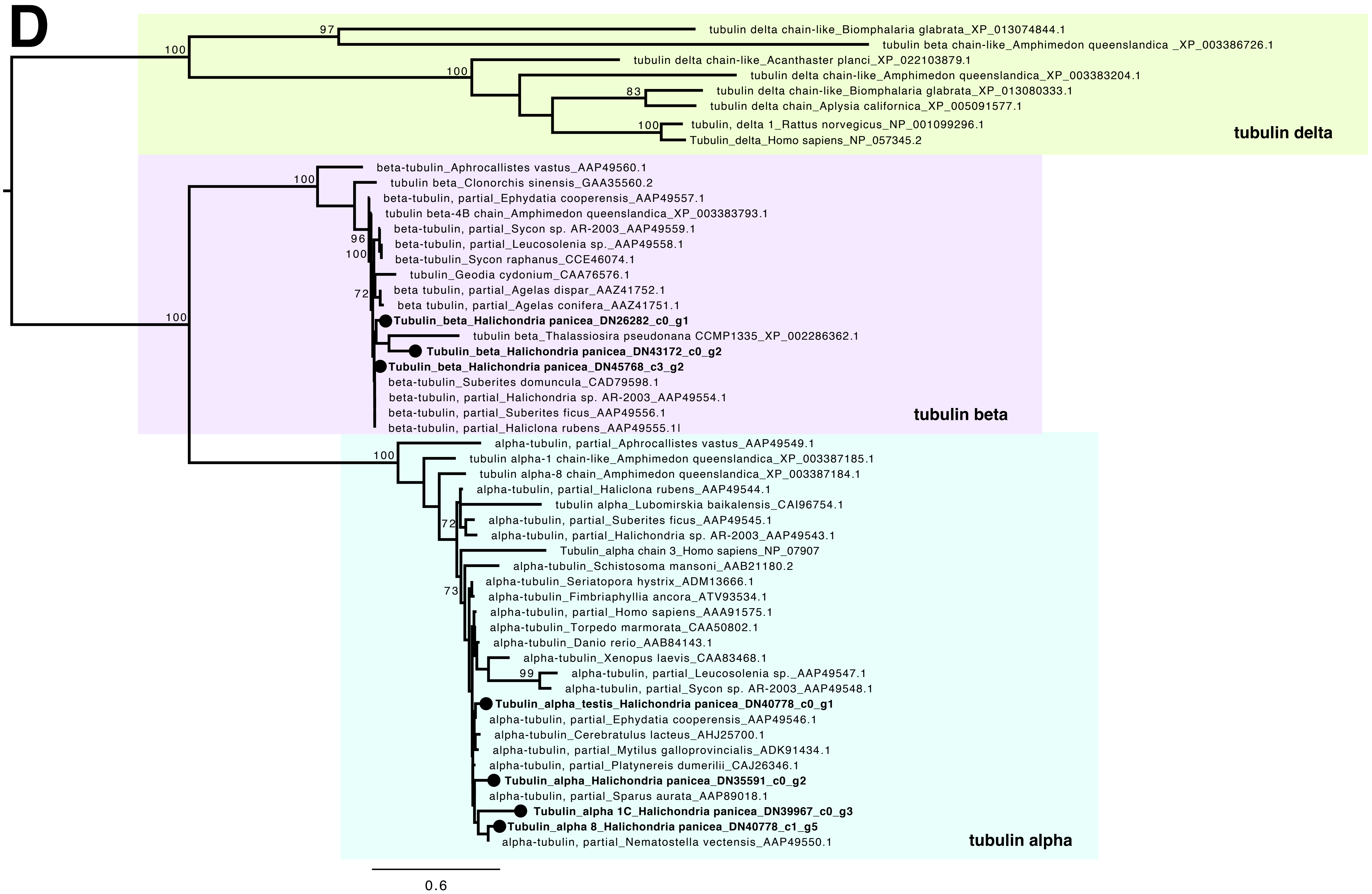
D
tubulin delta chain-like_Biomphalaria glabrata_XP_013074844.1
tubulin beta chain-like_Amphimedon queenslandica _XP_003386726.1
tubulin delta chain-like_Acanthaster planci_XP_022103879.1
tubulin delta chain-like_Amphimedon queenslandica_XP_003383204.1
tubulin delta chain-like_Biomphalaria glabrata_XP_013080333.1
tubulin delta chain_Aplysia californica_XP_005091577.1
tubulin, delta 1_Rattus norvegicus_NP_001099296.1
Tubulin_delta_Homo sapiens_NP_057345.2
tubulin delta
beta-tubulin_Aphrocallistes vastus_AAP49560.1
tubulin beta_Clonorchis sinensis_GAA35560.2
beta-tubulin, partial_Ephydatia cooperensis_AAP49557.1
tubulin beta-4B chain_Amphimedon queenslandica_XP_003383793.1
beta-tubulin, partial_Sycon sp. AR-2003_AAP49559.1
beta-tubulin, partial_Leucosolenia sp._AAP49558.1
beta-tubulin_Sycon raphanus_CCE46074.1
tubulin_Geodia cydonium_CAA76576.1
beta tubulin, partial_Agelas dispar_AAZ41752.1
beta tubulin, partial_Agelas conifera_AAZ41751.1
Tubulin_beta_Halichondria panicea_DN26282_c0_g1
tubulin beta_Thalassiosira pseudonana CCMP1335_XP_002286362.1
Tubulin_beta_Halichondria panicea_DN43172_c0_g2
Tubulin_beta_Halichondria panicea_DN45768_c3_g2
beta-tubulin_Suberites domuncula_CAD79598.1
beta-tubulin, partial_Halichondria sp. AR-2003_AAP49554.1
beta-tubulin, partial_Suberites ficus_AAP49556.1
beta-tubulin, partial_Haliclona rubens_AAP49555.1l
tubulin beta
alpha-tubulin, partial_Aphrocallistes vastus_AAP49549.1
tubulin alpha-1 chain-like_Amphimedon queenslandica_XP_003387185.1
tubulin alpha-8 chain_Amphimedon queenslandica_XP_003387184.1
alpha-tubulin, partial_Haliclona rubens_AAP49544.1
tubulin alpha_Lubomirskia baikalensis_CAI96754.1
alpha-tubulin, partial_Suberites ficus_AAP49545.1
alpha-tubulin, partial_Halichondria sp. AR-2003_AAP49543.1
Tubulin_alpha chain 3_Homo sapiens_NP_07907
alpha-tubulin_Schistosoma mansoni_AAB21180.2
alpha-tubulin_Seriatopora hystrix_ADM13666.1
alpha-tubulin_Fimbriaphyllia ancora_ATV93534.1
alpha-tubulin, partial_Homo sapiens_AAA91575.1
alpha-tubulin_Torpedo marmorata_CAA50802.1
alpha-tubulin_Danio rerio_AAB84143.1
alpha-tubulin_Xenopus laevis_CAA83468.1
alpha-tubulin, partial_Leucosolenia sp._AAP49547.1
alpha-tubulin, partial_Sycon sp. AR-2003_AAP49548.1
Tubulin_alpha_testis_Halichondria panicea_DN40778_c0_g1
alpha-tubulin, partial_Ephydatia cooperensis_AAP49546.1
alpha-tubulin_Cerebratulus lacteus_AHJ25700.1
alpha-tubulin, partial_Mytilus galloprovincialis_ADK91434.1
alpha-tubulin, partial_Platynereis dumerilii_CAJ26346.1
Tubulin_alpha_Halichondria panicea_DN35591_c0_g2
alpha-tubulin, partial_Sparus aurata_AAP89018.1
Tubulin_alpha 1C_Halichondria panicea_DN39967_c0_g3
Tubulin_alpha 8_Halichondria panicea_DN40778_c1_g5
alpha-tubulin, partial_Nematostella vectensis_AAP49550.1
tubulin alpha
97
100
100
83
100
100
100
96
100
72
100
72
73
99
0.6
