# Supplementary material for: Molecular machineries of ciliogenesis, cell survival, and vasculogenesis are differentially expressed during regeneration in explants of the demosponge Halichondria panicea
Source: BMC Genomics. 2022 Dec 29;23:858. doi: 10.1186/s12864-022-09035-0 (PMC9798719; doi:10.1186/s12864-022-09035-0)
Supplement: Supplementary file 4 — Additional file 4. [file 12864_2022_9035_MOESM4_ESM.pdf]

Supplementary Figure 1. A. Library size (in number of reads) per sample. **B.** PCA of the final samples used in the differential expression analysis. **C.** Correlation matrix of the final samples used in the differential expression analysis. **D.** Enrichment analysis of the Gene Ontology (GO) terms per condition. **E.** Treemaps showing the GO categories associated to the differentially expressed genes in each condition (NOE and PE) obtained in REVIGO. **F.** Treemaps showing the GO categories associated to the differentially expressed genes in each condition (PA and PE) obtained in REVIGO. **G–J.** Gene networks showing the connections between the differentially expressed genes in each condition.

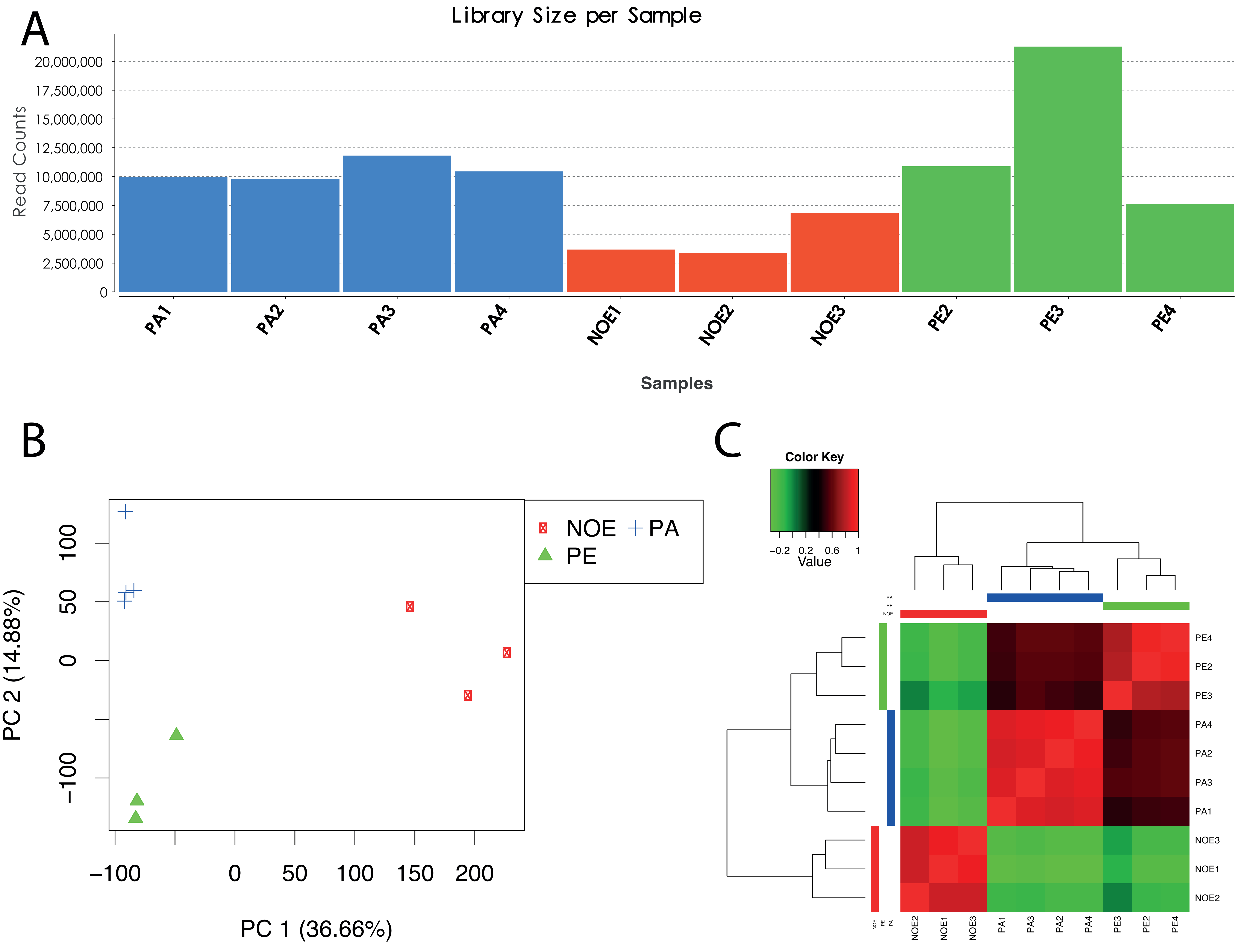

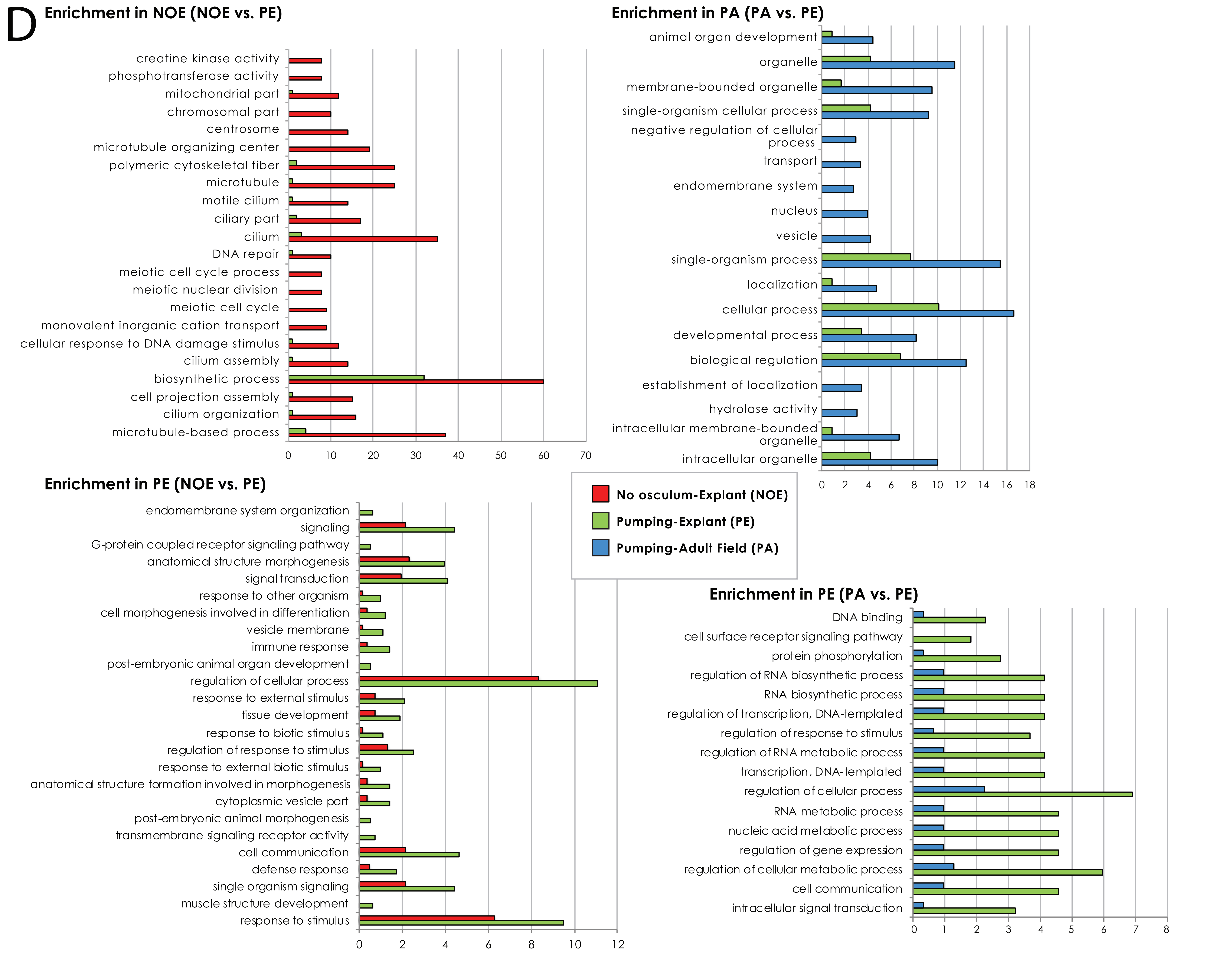
D
Enrichment in NOE (NOE vs. PE)
creatine kinase activity
phosphotransferase activity
mitochondrial part
chromosomal part
centrosome
microtubule organizing center
polymeric cytoskeletal fiber
microtubule
motile cilium
ciliary part
cilium
DNA repair
meiotic cell cycle process
meiotic nuclear division
meiotic cell cycle
monovalent inorganic cation transport
cellular response to DNA damage stimulus
cilium assembly
biosynthetic process
cell projection assembly
cilium organization
microtubule-based process
0 10 20 30 40 50 60 70
Enrichment in PA (PA vs. PE)
animal organ development
organelle
membrane-bounded organelle
single-organism cellular process
negative regulation of cellular process
transport
endomembrane system
nucleus
vesicle
single-organism process
localization
cellular process
developmental process
biological regulation
establishment of localization
hydrolase activity
intracellular membrane-bounded organelle
intracellular organelle
0 2 4 6 8 10 12 14 16 18
Enrichment in PE (NOE vs. PE)
endomembrane system organization
signaling
G-protein coupled receptor signaling pathway
anatomical structure morphogenesis
signal transduction
response to other organism
cell morphogenesis involved in differentiation
vesicle membrane
immune response
post-embryonic animal organ development
regulation of cellular process
response to external stimulus
tissue development
response to biotic stimulus
regulation of response to stimulus
response to external biotic stimulus
anatomical structure formation involved in morphogenesis
cytoplasmic vesicle part
post-embryonic animal morphogenesis
transmembrane signaling receptor activity
cell communication
defense response
single organism signaling
muscle structure development
response to stimulus
0 2 4 6 8 10 12
No osculum-Explant (NOE)
Pumping-Explant (PE)
Pumping-Adult Field (PA)
Enrichment in PE (PA vs. PE)
DNA binding
cell surface receptor signaling pathway
protein phosphorylation
regulation of RNA biosynthetic process
RNA biosynthetic process
regulation of transcription, DNA-templated
regulation of response to stimulus
regulation of RNA metabolic process
transcription, DNA-templated
regulation of cellular process
RNA metabolic process
nucleic acid metabolic process
regulation of gene expression
regulation of cellular metabolic process
cell communication
intracellular signal transduction
0 1 2 3 4 5 6 7 8

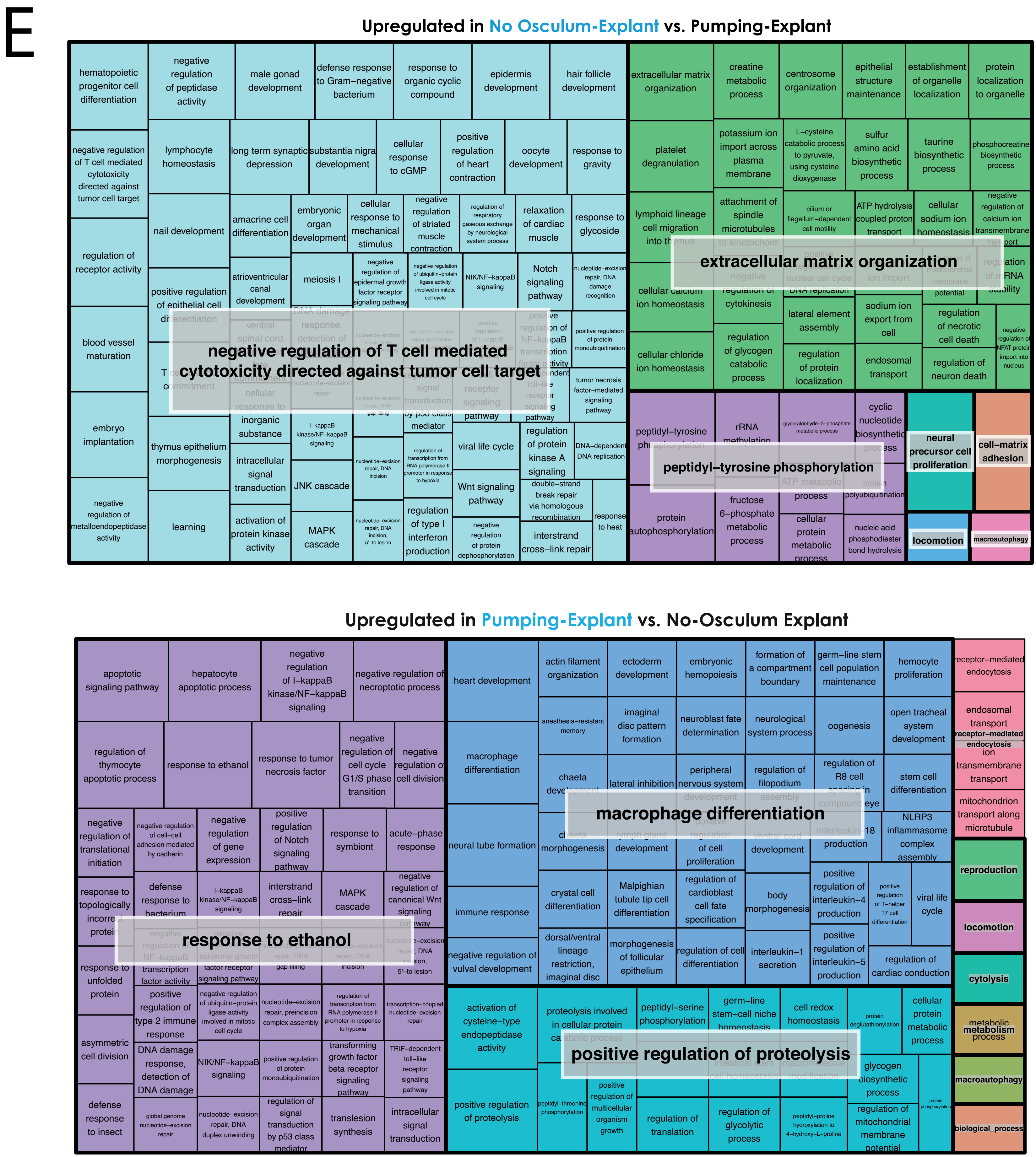

E
Upregulated in No Osculum-Explant vs. Pumping-Explant
hematopoietic progenitor cell differentiation
negative regulation of peptidase activity
male gonad development
defense response to Gram−negative bacterium
response to organic cyclic compound
epidermis development
hair follicle development
extracellular matrix organization
creatine metabolic process
centrosome organization
epithelial structure maintenance
establishment of organelle localization
protein localization to organelle
negative regulation of T cell mediated cytotoxicity directed against tumor cell target
lymphocyte homeostasis
long term synaptic depression
substantia nigra development
cellular response to cGMP
positive regulation of heart contraction
oocyte development
response to gravity
platelet degranulation
potassium ion import across plasma membrane
L−cysteine catabolic process to pyruvate, using cysteine dioxygenase
sulfur amino acid biosynthetic process
taurine biosynthetic process
phosphocreatine biosynthetic process
nail development
amacrine cell differentiation
embryonic organ development
cellular response to mechanical stimulus
negative regulation of striated muscle contraction
regulation of respiratory gaseous exchange by neurological system process
relaxation of cardiac muscle
response to glycoside
lymphoid lineage cell migration into thymus
attachment of spindle microtubules to kinetochore
cilium or flagellum−dependent cell motility
ATP hydrolysis coupled proton transport
cellular sodium ion homeostasis
negative regulation of calcium ion transmembrane transport
regulation of receptor activity
positive regulation of epithelial cell differentiation
atrioventricular canal development
meiosis I
negative regulation of epidermal growth factor receptor signaling pathway
negative regulation of ubiquitin−protein ligase activity involved in mitotic cell cycle
NIK/NF−kappaB signaling
Notch signaling pathway
nucleotide−excision repair, DNA damage recognition
extracellular matrix organization
regulation of mRNA stability
cellular calcium ion homeostasis
regulation of cytokinesis
lateral element assembly
sodium ion export from cell
regulation of necrotic cell death
negative regulation of NFAT protein import into nucleus
blood vessel maturation
T cell commitment
negative regulation of T cell mediated cytotoxicity directed against tumor cell target
positive regulation of protein monoubiquitination
cellular chloride ion homeostasis
regulation of glycogen catabolic process
regulation of protein localization
endosomal transport
regulation of neuron death
tumor necrosis factor−mediated signaling pathway
embryo implantation
thymus epithelium morphogenesis
response to inorganic substance
I−kappaB kinase/NF−kappaB signaling
mediator
regulation of protein kinase A signaling
DNA−dependent DNA replication
viral life cycle
peptidyl−tyrosine phosphorylation
rRNA methylation
glyceraldehyde−3−phosphate metabolic process
cyclic nucleotide biosynthetic process
neural precursor cell proliferation
cell−matrix adhesion
intracellular signal transduction
JNK cascade
nucleotide−excision repair, DNA incision
regulation of transcription from RNA polymerase II promoter in response to hypoxia
Wnt signaling pathway
double−strand break repair via homologous recombination
ATP metabolic process
protein polyubiquitination
negative regulation of metalloendopeptidase activity
learning
activation of protein kinase activity
MAPK cascade
nucleotide−excision repair, DNA incision, 5'−to lesion
regulation of type I interferon production
negative regulation of protein dephosphorylation
interstrand cross−link repair
response to heat
protein autophosphorylation
fructose 6−phosphate metabolic process
cellular protein metabolic process
nucleic acid phosphodiester bond hydrolysis
locomotion
macroautophagy
Upregulated in Pumping-Explant vs. No-Osculum Explant
apoptotic signaling pathway
hepatocyte apoptotic process
negative regulation of I−kappaB kinase/NF−kappaB signaling
negative regulation of necroptotic process
heart development
actin filament organization
ectoderm development
embryonic hemopoiesis
formation of a compartment boundary
germ−line stem cell population maintenance
hemocyte proliferation
receptor−mediated endocytosis
regulation of thymocyte apoptotic process
response to ethanol
response to tumor necrosis factor
negative regulation of cell cycle G1/S phase transition
negative regulation of cell division
macrophage differentiation
anesthesia−resistant memory
imaginal disc pattern formation
neuroblast fate determination
neurological system process
oogenesis
open tracheal system development
endosomal transport
receptor−mediated endocytosis
ion transmembrane transport
chaeta development
lateral inhibition
peripheral nervous system development
regulation of filopodium assembly
regulation of R8 cell spacing in compound eye
stem cell differentiation
macrophage differentiation
mitochondrion transport along microtubule
negative regulation of translational initiation
negative regulation of cell−cell adhesion mediated by cadherin
negative regulation of gene expression
positive regulation of Notch signaling pathway
response to symbiont
acute−phase response
neural tube formation
chaeta morphogenesis
lymph gland development
positive regulation of cell proliferation
ventral cord development
interleukin−18 production
NLRP3 inflammasome complex assembly
reproduction
response to topologically incorrect protein
defense response to bacterium
I−kappaB kinase/NF−kappaB signaling
interstrand cross−link repair
MAPK cascade
negative regulation of canonical Wnt signaling pathway
crystal cell differentiation
Malpighian tubule tip cell differentiation
regulation of cardioblast cell fate specification
body morphogenesis
positive regulation of interleukin−4 production
positive regulation of T−helper 17 cell differentiation
viral life cycle
immune response
locomotion
response to ethanol
response to unfolded protein
NF−kappaB transcription factor activity
factor receptor signaling pathway
nucleotide−excision repair, DNA incision, 5'−to lesion
negative regulation of vulval development
dorsal/ventral lineage restriction, imaginal disc
morphogenesis of follicular epithelium
regulation of cell differentiation
interleukin−1 secretion
positive regulation of interleukin−5 production
regulation of cardiac conduction
cytolysis
positive regulation of type 2 immune response
negative regulation of ubiquitin−protein ligase activity involved in mitotic cell cycle
nucleotide−excision repair, preincision complex assembly
regulation of transcription from RNA polymerase II promoter in response to hypoxia
transcription−coupled nucleotide−excision repair
activation of cysteine−type endopeptidase activity
proteolysis involved in cellular protein catabolic process
peptidyl−serine phosphorylation
germ−line stem−cell niche homeostasis
cell redox homeostasis
protein deglutathionylation
cellular protein metabolic process
metabolic process
metabolism
asymmetric cell division
DNA damage response, detection of DNA damage
NIK/NF−kappaB signaling
positive regulation of protein monoubiquitination
transforming growth factor beta receptor signaling pathway
TRIF−dependent toll−like receptor signaling pathway
positive regulation of proteolysis
intestinal stem cell homeostasis
macromolecule modification
glycogen biosynthetic process
macroautophagy
defense response to insect
global genome nucleotide−excision repair
nucleotide−excision repair, DNA duplex unwinding
regulation of signal transduction by p53 class mediator
translesion synthesis
intracellular signal transduction
positive regulation of proteolysis
peptidyl−threonine phosphorylation
positive regulation of multicellular organism growth
regulation of translation
regulation of glycolytic process
peptidyl−proline hydroxylation to 4−hydroxy−L−proline
regulation of mitochondrial membrane potential
protein phosphorylation
biological_process

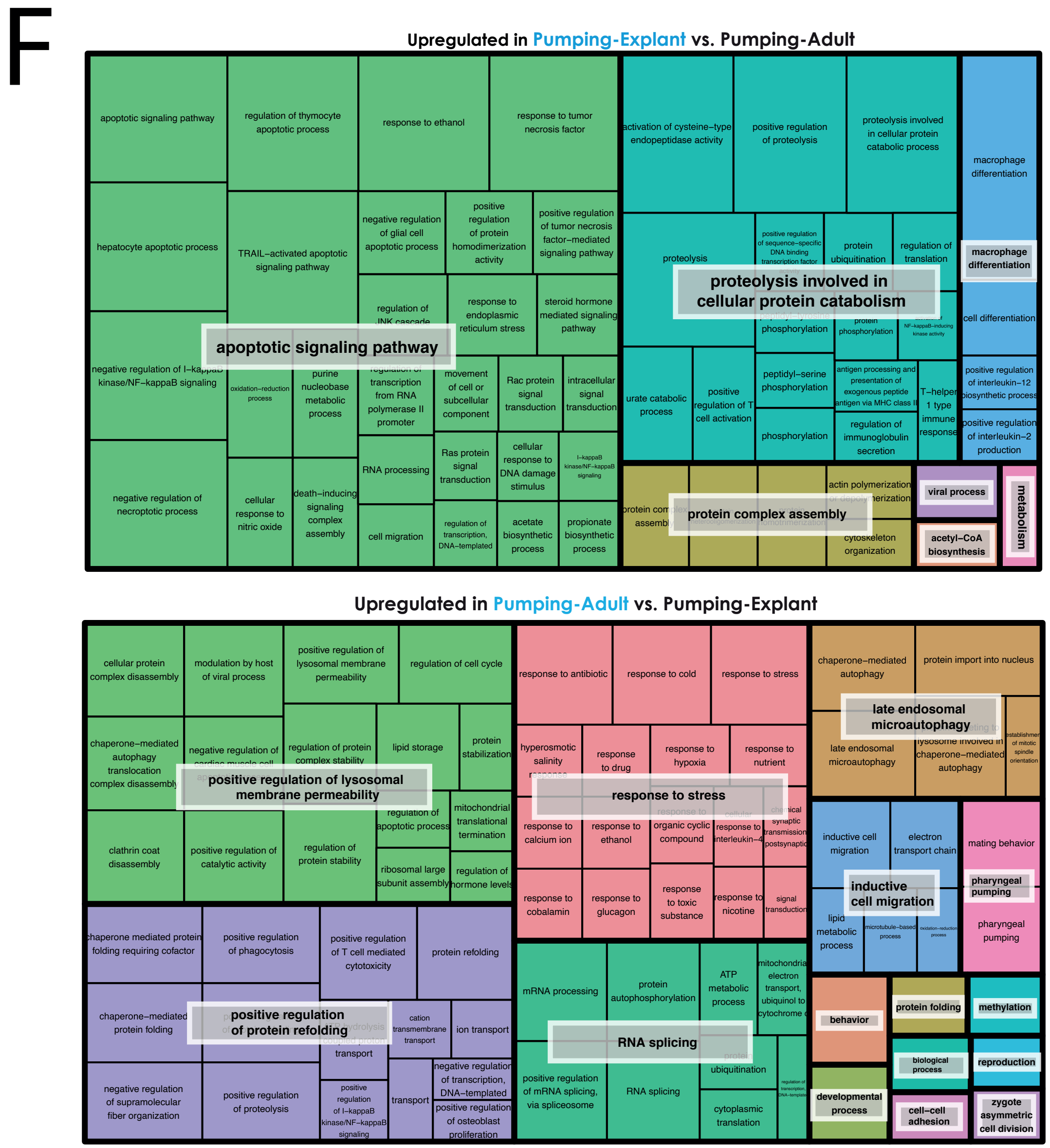
F
Upregulated in Pumping-Explant vs. Pumping-Adult
apoptotic signaling pathway
regulation of thymocyte apoptotic process
response to ethanol
response to tumor necrosis factor
hepatocyte apoptotic process
TRAIL–activated apoptotic signaling pathway
negative regulation of glial cell apoptotic process
positive regulation of protein homodimerization activity
positive regulation of tumor necrosis factor–mediated signaling pathway
response to endoplasmic reticulum stress
steroid hormone mediated signaling pathway
negative regulation of I–kappaB kinase/NF–kappaB signaling
oxidation–reduction process
purine nucleobase metabolic process
movement of cell or subcellular component
Rac protein signal transduction
intracellular signal transduction
RNA processing
Ras protein signal transduction
cellular response to DNA damage stimulus
I–kappaB kinase/NF–kappaB signaling
negative regulation of necroptotic process
cellular response to nitric oxide
death–inducing signaling complex assembly
cell migration
regulation of transcription, DNA–templated
acetate biosynthetic process
propionate biosynthetic process
activation of cysteine–type endopeptidase activity
positive regulation of proteolysis
proteolysis involved in cellular protein catabolic process
macrophage differentiation
proteolysis
protein ubiquitination
regulation of translation
proteolysis involved in cellular protein catabolism
cell differentiation
urate catabolic process
positive regulation of T cell activation
peptidyl–serine phosphorylation
phosphorylation
antigen processing and presentation of exogenous peptide antigen via MHC class II
T–helper 1 type immune response
regulation of immunoglobulin secretion
positive regulation of interleukin–12 biosynthetic process
positive regulation of interleukin–2 production
actin polymerization or depolymerization
protein complex assembly
cytoskeleton organization
viral process
acetyl–CoA biosynthesis
metabolism
Upregulated in Pumping-Adult vs. Pumping-Explant
cellular protein complex disassembly
modulation by host of viral process
positive regulation of lysosomal membrane permeability
regulation of cell cycle
chaperone–mediated autophagy translocation complex disassembly
negative regulation of cardiac muscle cell apoptotic process
regulation of protein complex stability
lipid storage
protein stabilization
positive regulation of lysosomal membrane permeability
regulation of apoptotic process
mitochondrial translational termination
clathrin coat disassembly
positive regulation of catalytic activity
regulation of protein stability
ribosomal large subunit assembly
regulation of hormone levels
chaperone mediated protein folding requiring cofactor
positive regulation of phagocytosis
positive regulation of T cell mediated cytotoxicity
protein refolding
chaperone–mediated protein folding
positive regulation of protein refolding
cation transmembrane transport
ion transport
transport
negative regulation of supramolecular fiber organization
positive regulation of proteolysis
positive regulation of I–kappaB kinase/NF–kappaB signaling
negative regulation of transcription, DNA–templated
positive regulation of osteoblast proliferation
response to antibiotic
response to cold
response to stress
hyperosmotic salinity response
response to drug
response to hypoxia
response to nutrient
response to stress
response to calcium ion
response to ethanol
response to organic cyclic compound
response to interleukin–4
chemical synaptic transmission, postsynaptic
response to cobalamin
response to glucagon
response to toxic substance
response to nicotine
signal transduction
mRNA processing
protein autophosphorylation
ATP metabolic process
mitochondrial electron transport, ubiquinol to cytochrome c
RNA splicing
protein ubiquitination
positive regulation of mRNA splicing, via spliceosome
cytoplasmic translation
chaperone–mediated autophagy
protein import into nucleus
late endosomal microautophagy
lysosome involved in chaperone–mediated autophagy
establishment of mitotic spindle orientation
inductive cell migration
electron transport chain
mating behavior
pharyngeal pumping
lipid metabolic process
microtubule–based process
oxidation–reduction process
behavior
protein folding
methylation
biological process
reproduction
developmental process
cell–cell adhesion
zygote asymmetric cell division

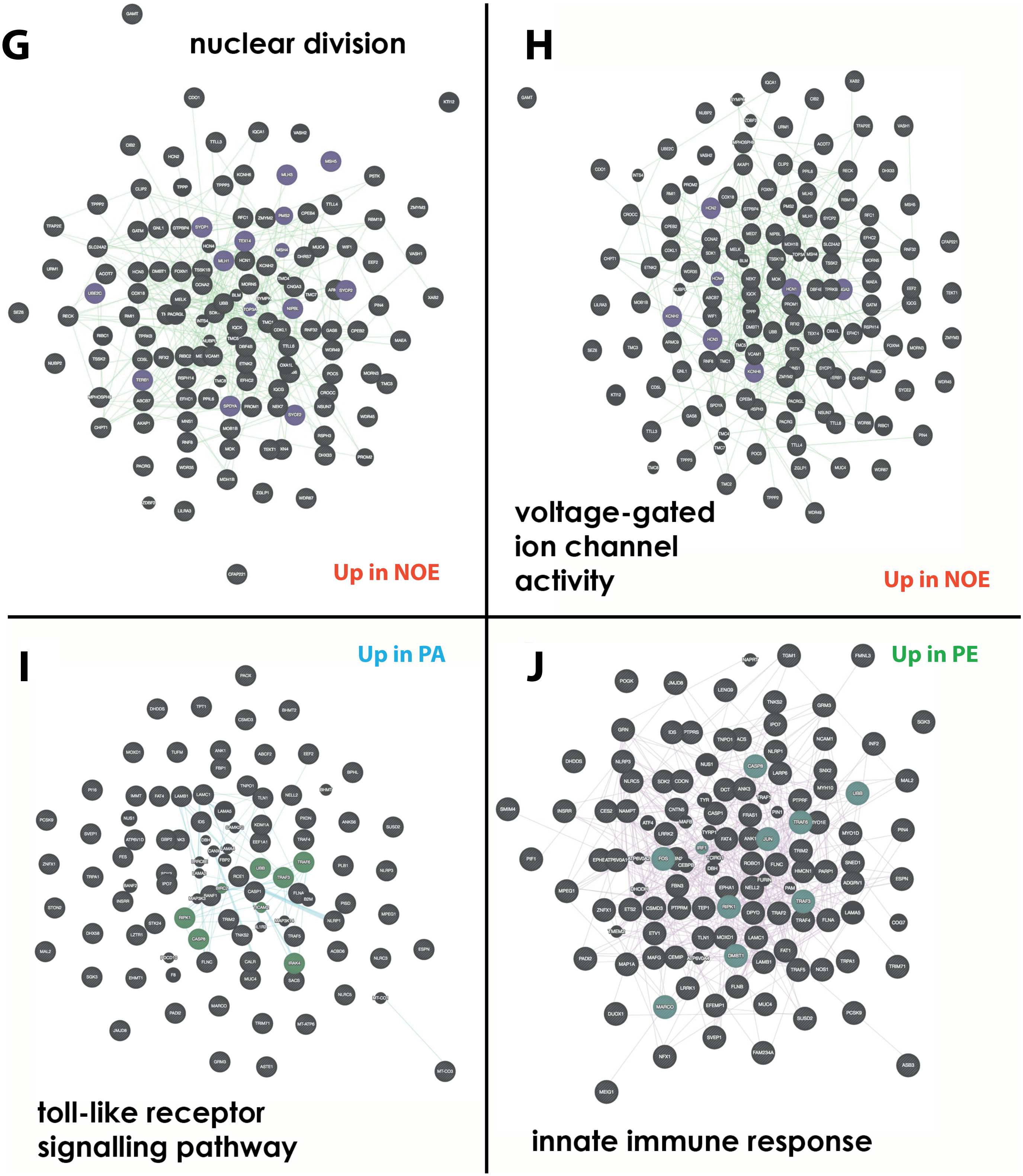

G
nuclear division
Up in NOE
H
voltage-gated ion channel activity
Up in NOE
I
Up in PA
toll-like receptor signalling pathway
J
Up in PE
innate immune response
