# Supplementary material for: Molecular machineries of ciliogenesis, cell survival, and vasculogenesis are differentially expressed during regeneration in explants of the demosponge Halichondria panicea
Source: BMC Genomics. 2022 Dec 29;23:858. doi: 10.1186/s12864-022-09035-0 (PMC9798719; doi:10.1186/s12864-022-09035-0)

Supplementary Figure 2. A. PCA of the original samples used for the assembly. **B.** Heatmap showing the differentially expressed genes and clustering of samples when using the complete set of samples.

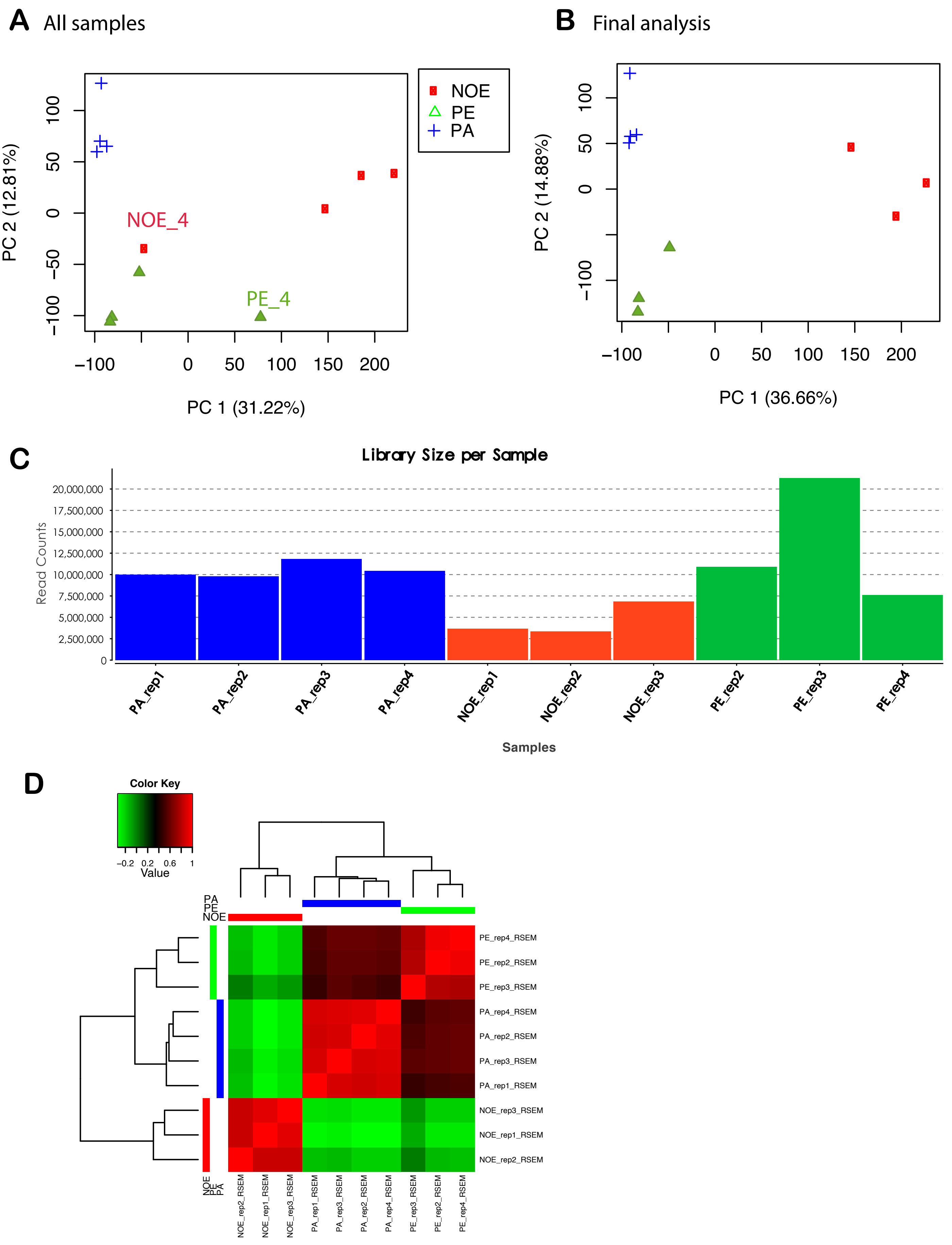

Supplement: Supplementary file 5 — Additional file 5. [file 12864_2022_9035_MOESM5_ESM.pdf]
