# Supplementary material for: Molecular machineries of ciliogenesis, cell survival, and vasculogenesis are differentially expressed during regeneration in explants of the demosponge Halichondria panicea
Source: BMC Genomics. 2022 Dec 29;23:858. doi: 10.1186/s12864-022-09035-0 (PMC9798719; doi:10.1186/s12864-022-09035-0)

Supplementary Figure 4. Differentially expressed genes related to ciliogenesis, stress, contractility, and sensory functions. GO terms are shown on the left side with parent categories on the right. The detailed structure of the flagellum is shown on the right side of the figure.

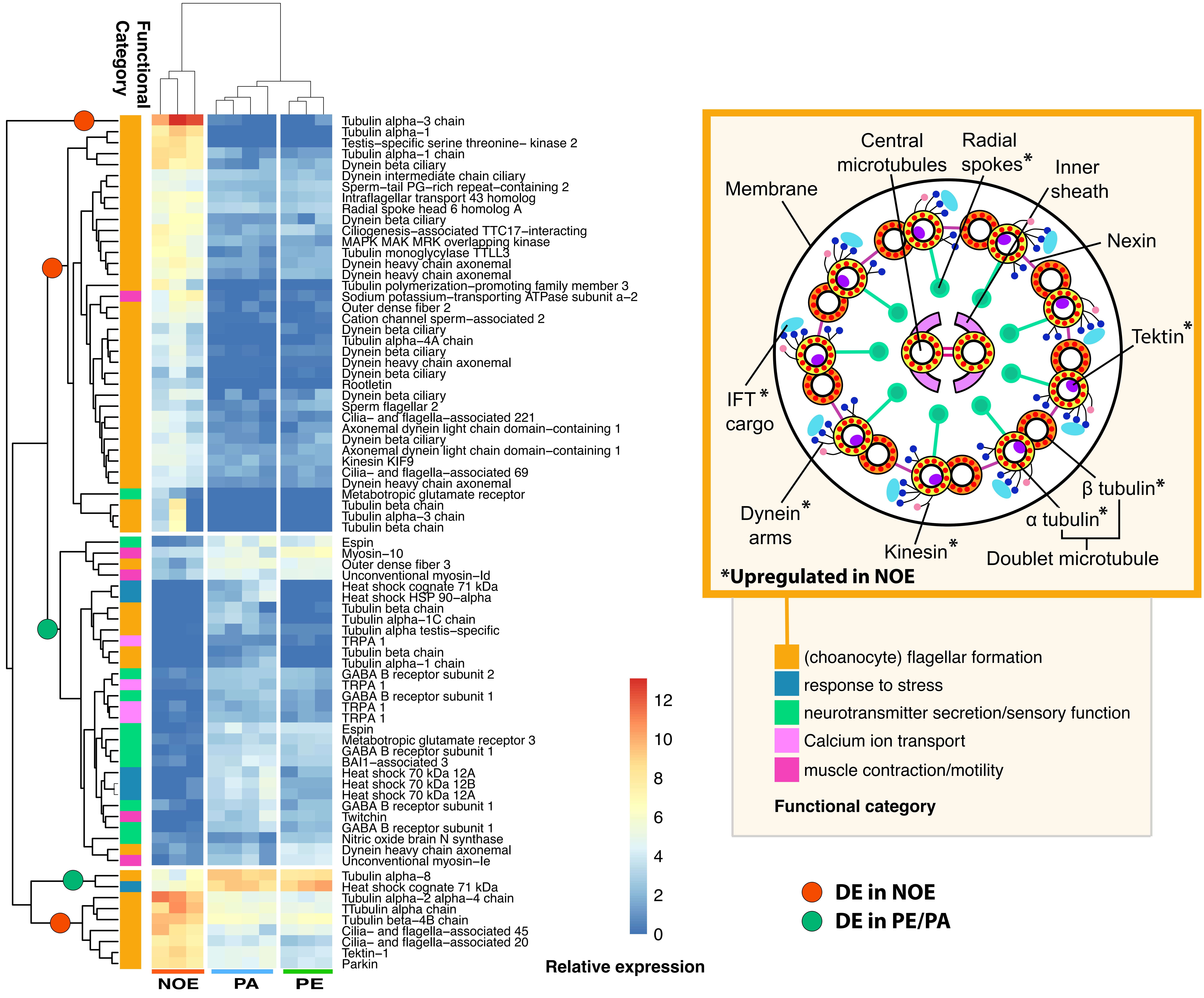

Supplement: Supplementary file 7 — Additional file 7. [file 12864_2022_9035_MOESM7_ESM.pdf]
